# Microsampling tools for collecting, processing, and storing blood at the point‐of‐care

**DOI:** 10.1002/btm2.10476

**Published:** 2022-12-29

**Authors:** Keith R. Baillargeon, Charles R. Mace

**Affiliations:** ^1^ Department of Chemistry, Laboratory for Living Devices Tufts University Medford Massachusetts USA

**Keywords:** dried blood spot, microsampling, plasma separation, point‐of‐care, quality, volumetric

## Abstract

In the wake of the COVID‐19 global pandemic, self‐administered microsampling tools have reemerged as an effective means to maintain routine healthcare assessments without inundating hospitals or clinics. Finger‐stick collection of blood is easily performed at home, in the workplace, or at the point‐of‐care, obviating the need for a trained phlebotomist. While the initial collection of blood is facile, the diagnostic or clinical utility of the sample is dependent on how the sample is processed and stored prior to transport to an analytical laboratory. The past decade has seen incredible innovation for the development of new materials and technologies to collect low‐volume samples of blood with excellent precision that operate independently of the hematocrit effect. The final application of that blood (i.e., the test to be performed) ultimately dictates the collection and storage approach as certain materials or chemical reagents can render a sample diagnostically useless. Consequently, there is not a single microsampling tool that is capable of addressing every clinical need at this time. In this review, we highlight technologies designed for patient‐centric microsampling blood at the point‐of‐care and discuss their utility for quantitative sampling as a function of collection material and technique. In addition to surveying methods for collecting and storing whole blood, we emphasize the need for direct separation of the cellular and liquid components of blood to produce cell‐free plasma to expand clinical utility. Integrating advanced functionality while maintaining simple user operation presents a viable means of revolutionizing self‐administered microsampling, establishing new avenues for innovation in materials science, and expanding access to healthcare.

## 1 INTRODUCTION

Healthcare systems generally operate in a reactive manner: when an individual experiences an abnormal symptom, they react by seeking medical attention. To confirm a diagnosis, healthcare professionals collect biological samples that are sent out to a laboratory for testing, and days to weeks can lapse before the results are obtained to administer a treatment regimen for the patient. If an individual lacks apparent symptoms, they may suffer from a medical condition without the ability to identify the underlying cause for a substantial amount of time. This lapse in healthcare can be further exacerbated by challenges associated with testing at the point‐of‐care or in resource‐limited settings. As a result, a reactive healthcare system does not benefit patients who require immediate medical intervention to attend to their health issues. Instead, an active system that allows patients to play a role in monitoring their health status or treatment efficacy is desirable. Patient‐centric microsampling low volumes of biofluids present an opportunity for a more active healthcare system.

Among the available biofluids that can be collected for health evaluations—including tears, sweat, urine, mucous—blood offers the most complex and diagnostically valuable sample matrix. Blood comprises distinct populations of cells (e.g., erythrocytes, lymphocytes, granulocytes, monocytes, macrophages, and platelets) and a rich carrier fluid (e.g., plasma or serum) containing myriad proteins, ions, dissolved gases, nutrients, and waste products.^1^ Analyzing whole blood, or its component parts, can provide a full panel of health and wellness indices for evaluating the cardiovascular system, various organ functionalities, vitamin/mineral levels, and countless other parameters indicative of health status.^2^ Clinically established reference values based on patient age or sex aid in making a preliminary diagnosis or determining the stage of a disease. Blood sampling also offers a means to monitor the effectiveness of medications to guide proper treatment. For these reasons, blood is often thought of as the ideal specimen for evaluating the health status of a patient.

**FIGURE 1:**
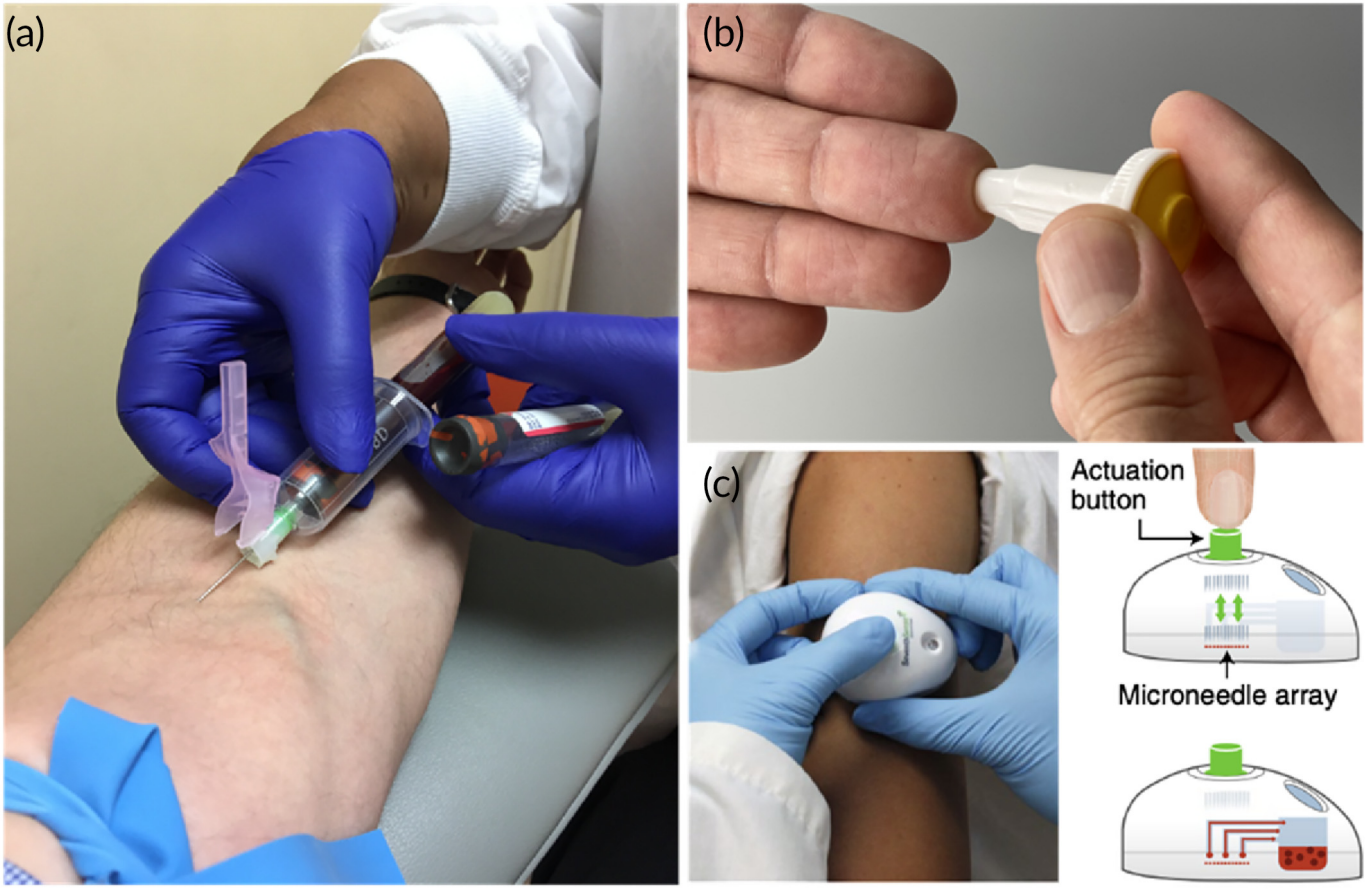
Techniques for generating blood samples. (a) Venipuncture collection (≥10 ml) performed by a trained phlebotomist (Matthew Lammers, CC BY‐SA 4.0, via Wikimedia Commons). (b) Self‐administered finger stick (≥10 μl) collection. (c) Capillary collection device, TAP (≥100 μl), by YourBio Health (Reprinted by permission from Springer Nature: Nature Biomedical Engineering, Ref 8, Copyright 2018).

Blood is routinely collected by venipuncture in centralized hospitals, local clinics, and even mobile clinics. In these settings, dedicated and highly trained staff are equipped with hypodermic needles, evacuated collection tubes (vacutainers) containing an anticoagulant, and a sharps container for safely disposing biohazardous materials following collection (Figure 1a).^3^ Inclusion of anticoagulant is critical to prevent clotting and maintain hematological indices prior to the analytical process. Collection volumes vary according to the tests ordered by the physician, but generally yield 2–10 ml per tube.^4^ While venipuncture offers a highly representative sample of circulating cells and analytical targets, this collection method is often invasive and painful for the patient.^5^ The associated large collection volumes can also accumulate a considerable source of waste depending on the final application. Additionally, venipuncture has the potential to expose phlebotomists to blood‐borne pathogens such as viruses (e.g., human immunodeficiency virus [HIV], HBV, HCV) and bacteria (e.g., *Treponema pallidum*, the pathogen that causes Syphilis) through needle‐stick.^5^ Due to the elevated risks associated with venipuncture for both patient and clinician, this method is not ideal for microsampling at the point‐of‐care.^6^

An alternative method for collecting smaller volumes of blood with minimized risk of exposure is capillary sampling via a finger stick (Figure 1b). In the simplest form, retractable single‐use lancets can be used to pierce a fingertip, heel, or earlobe to provide a range of expected volumes (e.g., low flow 10–20 μl; high flow 250–500 μl) dependent on needle gauge and penetration depth.^7^ Similar to collection by venipuncture, anticoagulants can be introduced to blood collected by finger stick using a pre‐coated capillary tube or storage of dried anticoagulant in a porous collection material. Advanced forms of capillary sampling also include microneedle array collection devices such as the TAP device by YourBio Health (Figure 1c), which yields an average of 104 ± 19 μl with just the push of a button.^8^ Finger‐stick collection represents an attractive method for self‐sampling that can be performed anywhere without the need for a trained clinician. Additionally, this method can result in less patient pain and discomfort in comparison to venipuncture, which can allow for more frequent sampling. While capillary sampling by finger stick offers advantages over venipuncture, different finger stick protocols can result in variations in critical blood parameters. Drop‐to‐drop variation was evaluated using a hematology analyzer for quantitation of hemoglobin and three‐point white blood cell (WBC) differential.^9^ A higher degree of variability was reported for hemoglobin, WBC count, and platelet count measurements from a finger stick compared to venous blood. However, the clinical utility of low volume finger stick sampling was reported within instrument variability for volumes equal to or greater than 60–100 μl. In this example, pooling multiple drops of blood from a finger stick provided a more representative sample compared to a single droplet of blood.

The total testing process can be divided into three main phases: (i) pre‐analytical (i.e., sample collection, handling, transportation, storage, and preparation); (ii) analytical (i.e., sample analysis); and (iii) post‐analytical (i.e., test validation, interpretation, and reporting). The Clinical Laboratory Improvement Amendments of 1988 regulates all clinical laboratory testing of biological samples for diagnostic, prevention, or treatment purposes.^10^ Criteria for acceptable performance is analyte dependent, but broadly states that coefficients of variation (%CV) should not exceed 20% of the target value, with some exceptions.^11^ For example, quantitation of hemoglobin (±4%), albumin (±8%), platelet count (±25%), and Troponin I (±30%) demonstrate the range of acceptable performance metrics as published by the Center for Medicare and Medicaid Services in 2019. While these criteria are often evaluated and reported during the post‐analytical phase, the majority of lab errors occur during the pre‐analytical phase and are most frequently caused by hemolysis (40–70%), insufficient sample volume (10–20%), and undue clotting (5–10%).^12^ These factors represent an outstanding need for improved tools and methods for collection of biological samples. Specifically, the storage and transportation of blood samples represent two remaining challenges at the point‐of‐care.

Sample stability with respect to hematological parameters is largely dependent on storage temperature and length of time.^13^ The World Health Organization (WHO) recommends samples of whole blood should be stored between 4 and 8°C for a maximum of 24 h and cannot be frozen.^3^ To extend storage life, plasma or serum should be separated from the sample and stored at 4–8°C for up to 7 days or frozen (−20°C) for longer periods of time. Additional storage recommendations are available depending on the desired laboratory analyses.^14^ These cold‐chain storage restraints put a substantial burden on the collection of blood in remote areas and often result in samples being discarded. For applications where immediate testing is not possible, and cold‐chain storage is unavailable, preserving the stability of the sample at ambient conditions could navigate the remaining challenges for self‐sampling low volumes of blood at the point‐of‐care or in resource‐limited settings.

As complicated and bulky clinical instruments are replaced by user‐friendly and compact point‐of‐care technologies, the collection method and subsequent analytical quality of biofluid samples ultimately determines the clinical relevance of the test result. Recently, several comprehensive reviews have summarized advances in microsampling techniques with respect to therapeutic drug monitoring,^15^ biobanking,^16^ paper spray mass spectrometry,^17^ and lab‐on‐paper devices.^18^ Most technologies have been reviewed with respect to analyte‐ or technique‐specific metrics of performance such as limits of detection and dynamic range as informed by established guidelines for method validation related to quantitative bioanalysis.^19^ This end‐point method of analysis is typically determined by the analysis method or instrumentation and does not include critical aspects of device performance such as sample input or output volumes, time requirements, user steps, or sample purity. In this review, we analyze the key aspects of device performance as a function of device design and materials to aid in identifying the correct sampling tool for self‐sampling, separating, and storing blood outside of clinical settings for use at the point‐of‐care. Our search criteria were limited to articles published between 2010 and 2020. Distinction between final sample state (e.g., liquid vs. dry) was indicated for each evaluation. Technologies that require electricity or external equipment—such as syringe pumps—were excluded from this review. Additionally, we survey different methods for passive generation of cell‐free plasma and discuss the advantages and limitations of each approach with respect to plasma quality and separation efficiency. Identifying the correct collection tool for targeted downstream testing could address the major sources of analytical errors associated with health diagnostics.

## 2 COLLECTION AND STORAGE OF WHOLE BLOOD AT THE POINT‐OF‐CARE

Capillary microsampling offers precise and accurate blood volumes stored within an open‐ended glass tube often coated with anticoagulant. The tube is filled end‐to‐end by capillary action upon contact with pool of blood. The resultant blood specimen can be transferred to a plastic microcentrifuge tube or similar low volume container (e.g., microtainer,^20^ minivette point‐of‐care test^21^) for storage or immediately applied to a point‐of‐care test (e.g., lateral flow test [LFT]^22^ or hand‐held analyzer^23^). Addition of an aqueous solution or buffer to the container washes out the sample and effectively dilutes it to a working volume. Beyond the volumetric advantages of this technique, little is offered with respect to potential for automation or analyte stability. Ultimately, capillary microsampling produces a low volume sample of liquid blood, which requires specific cold‐chain storage conditions to maintain analyte stability similar to venipuncture.

**FIGURE 2:**
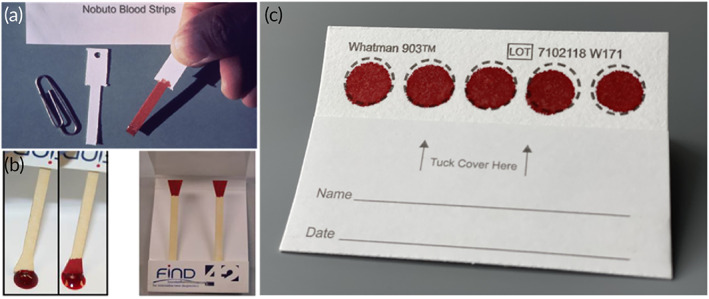
Whole blood collection technologies. (a) NOBUTO blood strips (Reprinted with permission from Ref 29). (b) Triangle paper dipsticks (Reprinted with permission from Ref 31). (c) Standard dried blood spot card (Whatman 903 protein saver). Each device is limited by unmetered sample absorption and the hematocrit effect.

In contrast to liquid samples, the power of storing dried blood in a porous matrix such as cellulosic or glass fiber materials—to maintain analyte stability in the absence of cold‐chain storage—has been extensively reported.^24^, ^25^ In fact, the stability of several analyte classes (e.g., drug metabolites, cytokines, and RNA) is improved upon drying as they are less susceptible to degradation by hydrolysis, photolytic processes, esterase, and RNAase activity.^26^, ^27^, ^28^ This technique has been demonstrated with two main form factors: (i) dipstick‐style paper strips and (ii) dried blood spot (DBS) cards (Table 1). NOBUTO blood collection strips comprise Advantex type 1 blood sampling paper and have been used for detection of avian influenza virus antibody in waterfowl (Figure 2a).^29^, ^30^ These dipstick‐style strips offer little in terms of handling protection to the user or drying considerations as these are essentially strips of raw filter paper. In contrast, the triangle paper dipstick comprises a laser cut Whatman GB003 filter paper inside a matchbook‐style case for improved handling and drying (Figure 2b).^31^ These strips are placed in contact with a volume of blood and allowed to wick until blood saturates the entire triangle‐shaped portion (approximately 20–40 μl) and allowed to dry while attached to the case. Similarly, DBS cards are traditionally affixed to a book‐style cardstock material for handling, drying, and recording patient information. DBS microsampling has been widely adopted for clinical use and offers several key advantages.^32^, ^33^

**TABLE 1:** Whole blood collection technologies

Technology	Input volume (μl)	Output volume (μl)	Application	Stability	References
NOBUTO strip	~40	~40	bELISA	25°C (3 months)	^29^
Triangle paper dipstick	21.1 ± 8.1%	21.1 ± 8.1%	LAMP	−20°C	^31^
DBS (Whatman 903)	70–100	70–100	ELISA	−20°C	^32^
DBS (Whatman 903)	20–30	NR	Protein extension assay	−20°C	^33^
VAPD	50–80	18.1 ± 0.7	HPLC	25°C (2 weeks)	^59^
VAPDmini	20–50	4.85 ± 0.15	HPLC	25°C (2 weeks)	^59^
HemaSpot HF	80	~80	LFT	25°C (1 month)	^60^
pDBS card	75	41.2 (10.3 per punch)	UV–vis, ICP‐AES, HPLC	NR	^63^
VAMS	10	10	LC–MS/MS	25°C (3 weeks)	^68^, ^69^
VAMS	10.6	10.6	LC–MS	NR	^72^
VAMS	20	20	LC–MS/MS	−20°C	^71^
VAMS	30	30	LC–MS/MS	25°C (2 months)	^70^
HemaPEN	20	10.96 (2.74 per punch)	LC–MS/MS	60°C (4 days)	^73^, ^74^
Hemaxis DB10	5.5	5.5	(SPE)‐LC–MS/MS	‐20°C	^75^
Hemaxis DB10	5–10	5–10	LC–MS	NR	^77^
Capitainer‐B	40	14.25 ± 3.0%	LC–MS/MS	NR	^80^
Capitainer‐B	35	13.5	LC–MS/MS	25°C (3 months) 60°C (4 days)	^78^
ADX test card	20–80	NR	NR	NR	^81^
Blood spheroids	30	30	Paper spray‐MS	25°C (28 days)	^83^

Abbreviations: DBS, dried blood spot; ELISA, enzyme‐linked immunosorbent assay; VAMS, volumetric absorptive microsampler.

Bang pioneered the use of filter paper for quantifying the concentration of glucose from DBS in 1913.^34^ Later in 1963, Guthrie and Susi described a simple screening method using DBS for detecting phenylketonuria in large populations of newborn infants.^35^ Since then, applications for DBS sampling have extended to over 2000 analytes^36^ including the analysis of small molecules,^37^ viral infections (e.g., HIV, HBV, HCV),^38^ therapeutic drug monitoring (e.g., antiepileptic drugs),^39^ anticancer drugs,^40^ and toxicology.^41^ Traditional DBS cards comprise a sample collection layer imprinted with circular zones to indicate sample addition affixed to a basic cardstock material for patient information (Figure 2c). Each zone accommodates approximately two to three drops of blood and each card typically contains five zones. Commercially available DBS cards are offered by various manufacturers for general protein analysis (e.g., Whatman 903 and Ahlstrom 226 protein saver cards)^42^ and specific nucleic acid stabilization (e.g., Whatman FTA DMPK‐A/B).^43^ Advantages of DBS cards for blood sampling include (i) ease of self‐sampling by direct finger stick, (ii) simplified transportation at ambient conditions, and (iii) ability to archive samples for retrospective analysis (i.e., biobanking).^44^, ^45^, ^46^ Additionally, DBS cards are generally inexpensive and require minimal equipment, which permits access to health‐related diagnostic data for hard‐to‐reach populations (i.e., remote settings) and surveillance efforts to monitor population level transmission of infection or track emerging disease. While the benefits of DBS cards for sampling blood at the point‐of‐care are myriad, they are limited by natural variations in patient hematocrit, which severely complicates quantitative analysis.

**FIGURE 3:**
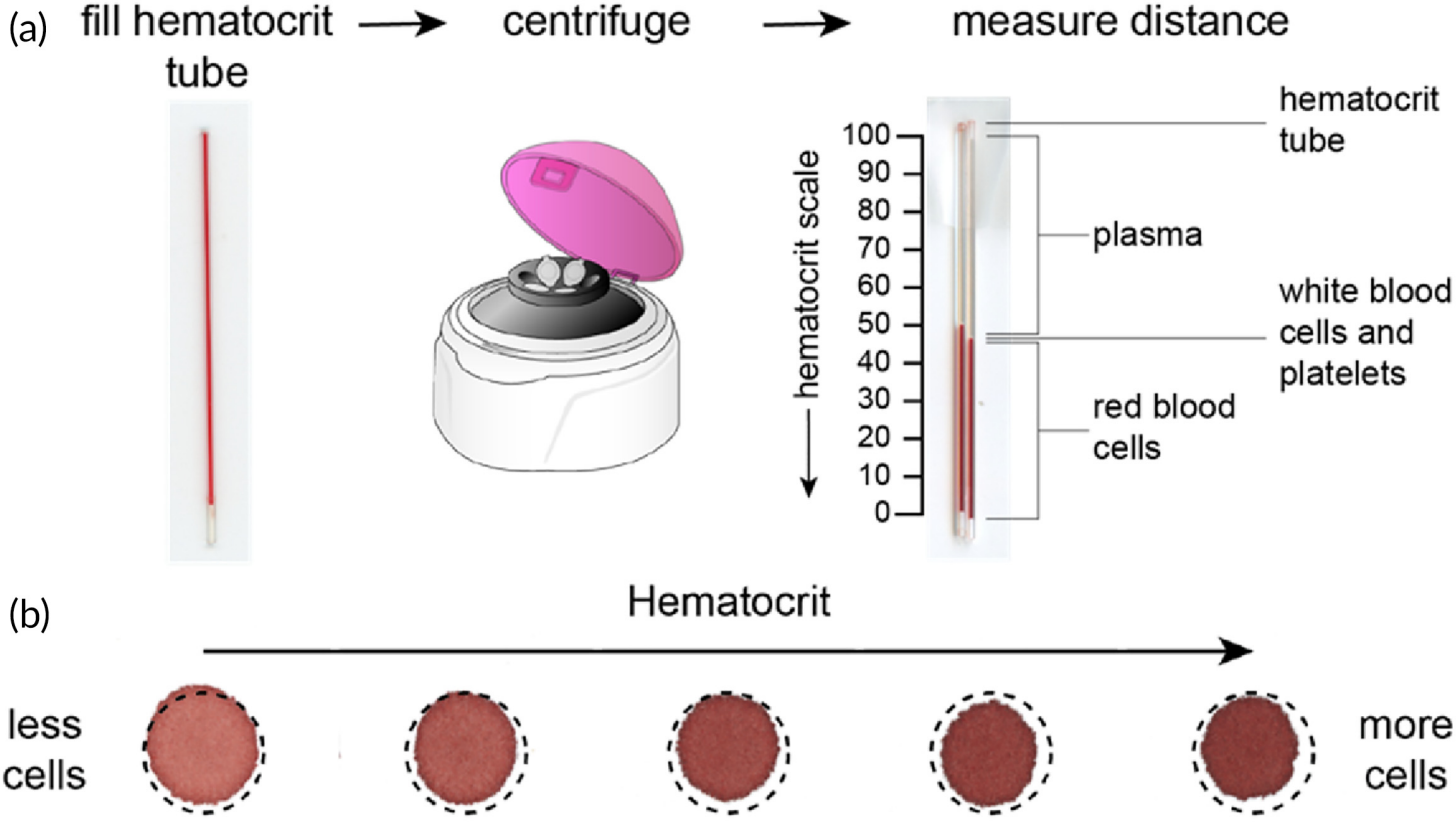
The hematocrit effect. (a) Blood samples are collected into a hematocrit microcapillary tube, centrifuged, and measured to report the ratio of RBC volume to total volume of blood by distance (Database Center for Life Science, CC BY 3.0, via Wikimedia Commons). (b) The hematocrit effect can be directly visualized by the reduced diameter of dried blood spot as a function of increased hematocrit value. Higher hematocrit samples contain a higher ratio of cells to plasma, which reduces wicking radius and leads to sampling bias from subpunch analysis (Adapted with permission from Ref 63, Copyright 2021 American Chemical Society).

**FIGURE 4:**
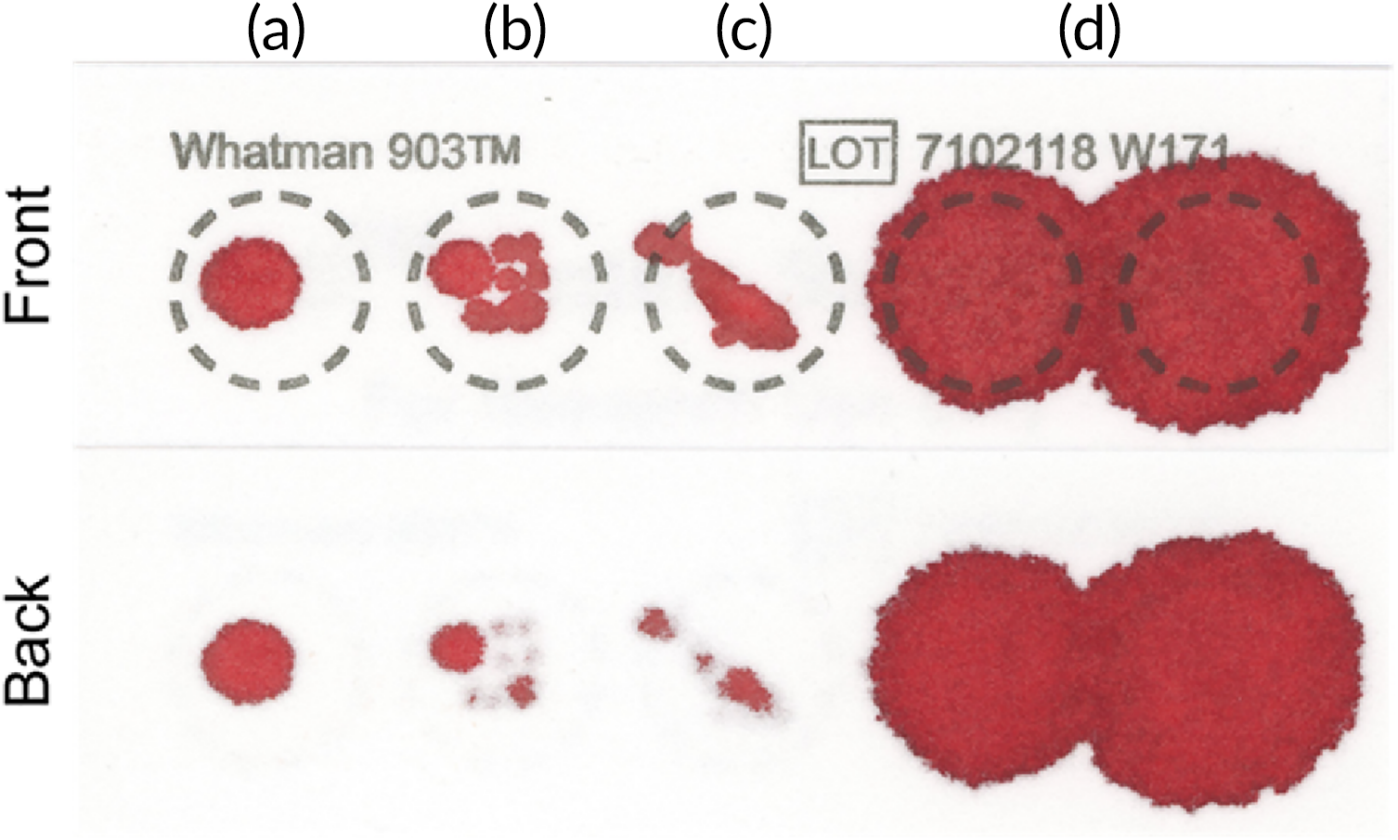
User‐errors associated with dried blood spot (DBS) sampling. Examples include (a) application of insufficient volume, (b) uneven sample application with multiple drops, (c) incomplete penetration through the thickness of the filter paper, and (d) coalescence of blood spots due to overfilling. Improper sampling can result in cards being discarded.

The hematocrit is described as the ratio of packed red blood cell (RBC) volume to the total volume of a blood sample following centrifugation (Figure 3a). Normal hematocrit values based on biological sex range from 36 to 48% for women and 41 to 50% for men. Additional factors such as age and hydration can affect an individual's hematocrit value.^47^ Changes in hematocrit can manifest a sampling bias in DBS cards due to inconsistent carrier fluid volume (i.e., liquid plasma) with respect to the quantity of cellular matter (i.e., RBCs, WBCs, and platelets).^48^, ^49^ This sampling bias can be further subdivided into (i) area bias and (ii) recovery bias (i.e., matrix effects).^50^ The area bias is largely driven by differences in viscosity related to the relative quantities of cells found in samples with different hematocrits. For example, a sample with a higher hematocrit will spread less than a sample with lower hematocrit, resulting in an inconsistent volume of sample contained in the same area of filter paper (i.e., subpunch) (Figure 3b). This area bias has been reported in multiple studies aimed at evaluating DBS cards.^51^, ^52^ The recovery bias is associated with uneven distribution, absorption, and higher evaporation rates of biofluid during the drying process, often resulting in nonhomogeneous blood spots.^53^ Both sources of sampling bias can be minimized or eliminated by employing a few basic strategies.^54^ Specifically, combining volumetric sample application with whole‐spot analysis will produce a precise volume of dried sample independent of the hematocrit value.^55^ Additionally, pairing whole‐spot analysis with an internal standard can help to address the recovery bias and account for loss of analyte to the extraction process.^56^ While these strategies seem simple, they are dependent on accurate volume application with the aid of a metered capillary tube or volumetric pipette, which limits use to trained staff.^57^ Poor sampling by DBS can typically be determined visually with respect to the outlined sample application zones (Figure 4).^58^ Here, DBS samples can be rejected for (i) insufficient volume (Figure 4a), uneven application (Figure 4b), incomplete saturation (Figure 4c), and overfilling (Figure 4d). Innovation in the field of self‐administered microsampling of blood aims to facilitate volumetric sampling for quantitative analysis without complicating the user‐experience. Various strategies have been described for restricting sample volume to minimize bias due to uncontrolled spreading through a porous material.

### 2.1 Volumetric dried blood sampling technologies

**FIGURE 5:**
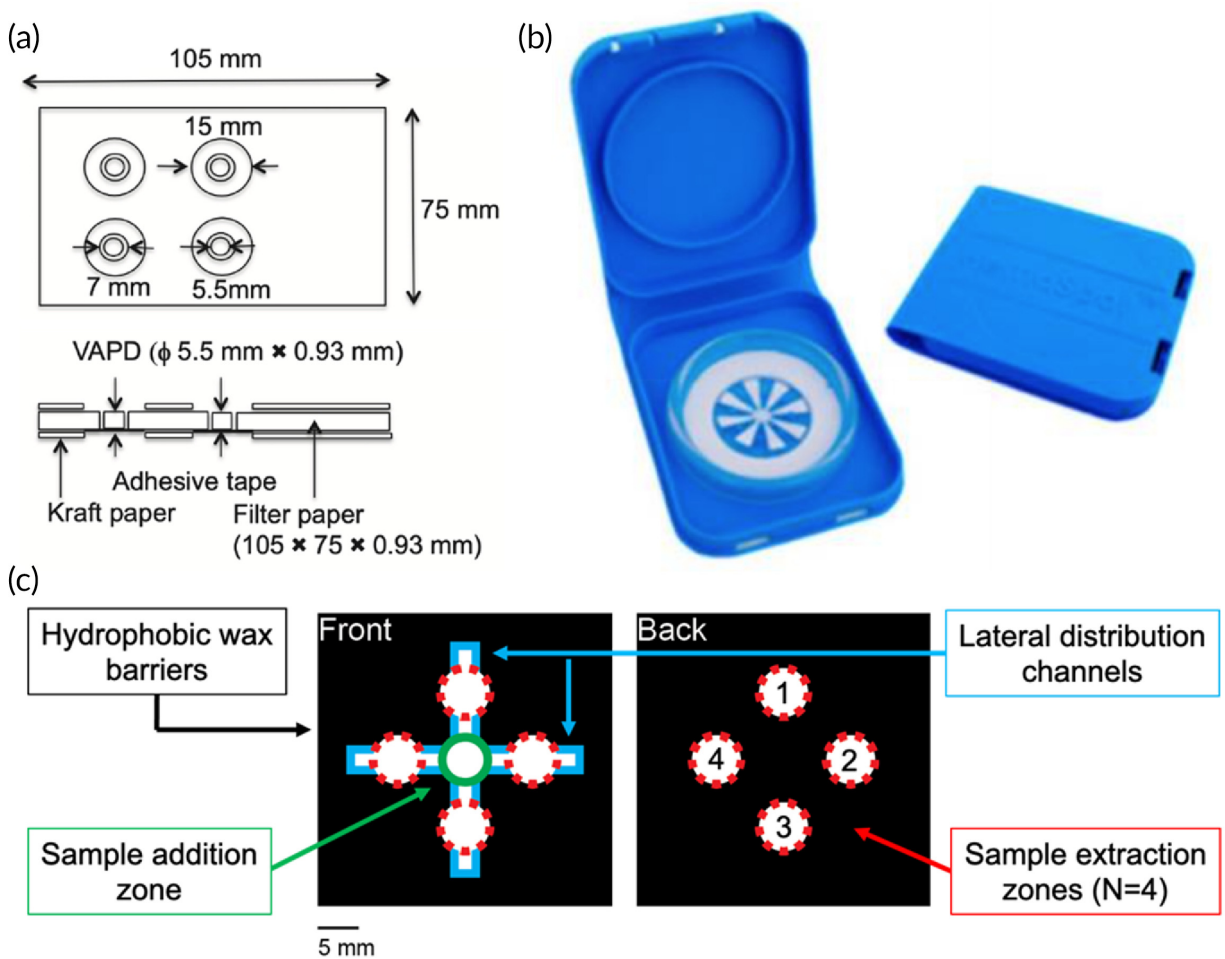
Volumetric dried blood spot (DBS) technologies. Each device operates independent of the hematocrit effect by restricting the area for sample absorption. (a) Volumetric absorptive paper disk (VAPD, Reprinted with permission from Ref 59) and (b) HemaSpot HF by Spot‐On Sciences (Ref 60, CC BY 4.0, via MDPI) precut the collection zones to restrict sample volume. (c) Patterned DBS (pDBS) cards use hydrophobic wax barriers to restrict sample flow and distribution (Adapted with permission from Ref 63, Copyright 2021 American Chemical Society).

The first strategy for volumetric sampling focuses on restricting the overall hydrophilic area, which absorbs and contains the final blood sample. The volumetric absorptive paper disk (VAPD) device comprises a precut paper disk surrounded by filter paper to eliminate the need for accurate volume application using pipettes or microcapillaries (Figure 5a).^59^ The precut disk (5.5 mm diameter) is positioned within a circular void in the filter paper using adhesive. The gap between the paper disk and the filter paper is small enough to allow excess blood to flow into the filter paper to ensure an accurate volume is contained within each disk. A similar device, termed VAPDmini, is functionally the same but on a smaller scale (3 mm diameter paper disk). Direct comparison of blood spots obtained using traditional DBS cards and VAPD devices yielded stark differences across hematocrit values. A 25 μl sample of blood (30–60% hematocrit) produced blood spots with different diameters on the top (6.7–6.0 mm) and bottom (6.8–4.7 mm) of a DBS card. The difference in calculated volume (assuming a truncated cone) using the measured diameters yielded 35.1–22.1 μl. In contrast, VAPD and VAPDmini operated independently of the hematocrit (20–70%) yielding 18.1 ± 1.2 μl and 4.85 ± 0.41 μl in a single precut disk, respectively. While these devices offered improved volumetric sampling compared to DBS cards, Nakahara et al. reported no difference in analyte recovery for clozapine and its metabolites by HPLC.

The HemaSpot HF (80 μl) and HD (140 μl) from Spot‐On Sciences were designed for finger‐stick sampling of blood emphasizing safe storage and transport at ambient temperature.^60^ The devices comprise eight precut wedges of absorbent paper connected at a center point, desiccant, and a tamper‐resistant cartridge with key (Figure 5b). Sample is applied to the center of the device for distribution to the paper wedges and dried by the surrounding desiccant. These devices were evaluated with plasma samples from dogs to detect parasitic disease (Visceral leishmaniasis)^61^ and human blood samples for HIV‐1 drug resistance testing.^62^ High sensitivity and specificity were achieved for dog plasma samples; however, genotyping was successful in only 67% of HemaSpots tested within a viral load range of 1000–100,000 copies/ml.

Quantitative sampling by volume restriction has also been achieved using traditional DBS filter papers impregnated with hydrophobic wax barriers to control blood flow and distribution.^63^ These patterned DBS (pDBS) cards comprise defined areas for (i) sample addition, (ii) lateral distribution, and (iii) four distinct extraction zones (Figure 5c). Physically restricting the sample enable complete saturation of the extraction zones with a sample input volume of 75 μl independent of the hematocrit (20–60%). The total sample output volume is approximately 41.2 μl (10.3 μl per zone) using a standard 6‐mm punch. Quantitation of hemoglobin yielded ≤7% error across the full range of hematocrit values, which represents a threefold improvement in accuracy using pDBS cards compared to unpatterned cards. pDBS cards offer a reproducible method for preparing and storing samples of blood with improved accuracy while maintaining current clinical protocols for processing DBS cards.

**FIGURE 6:**
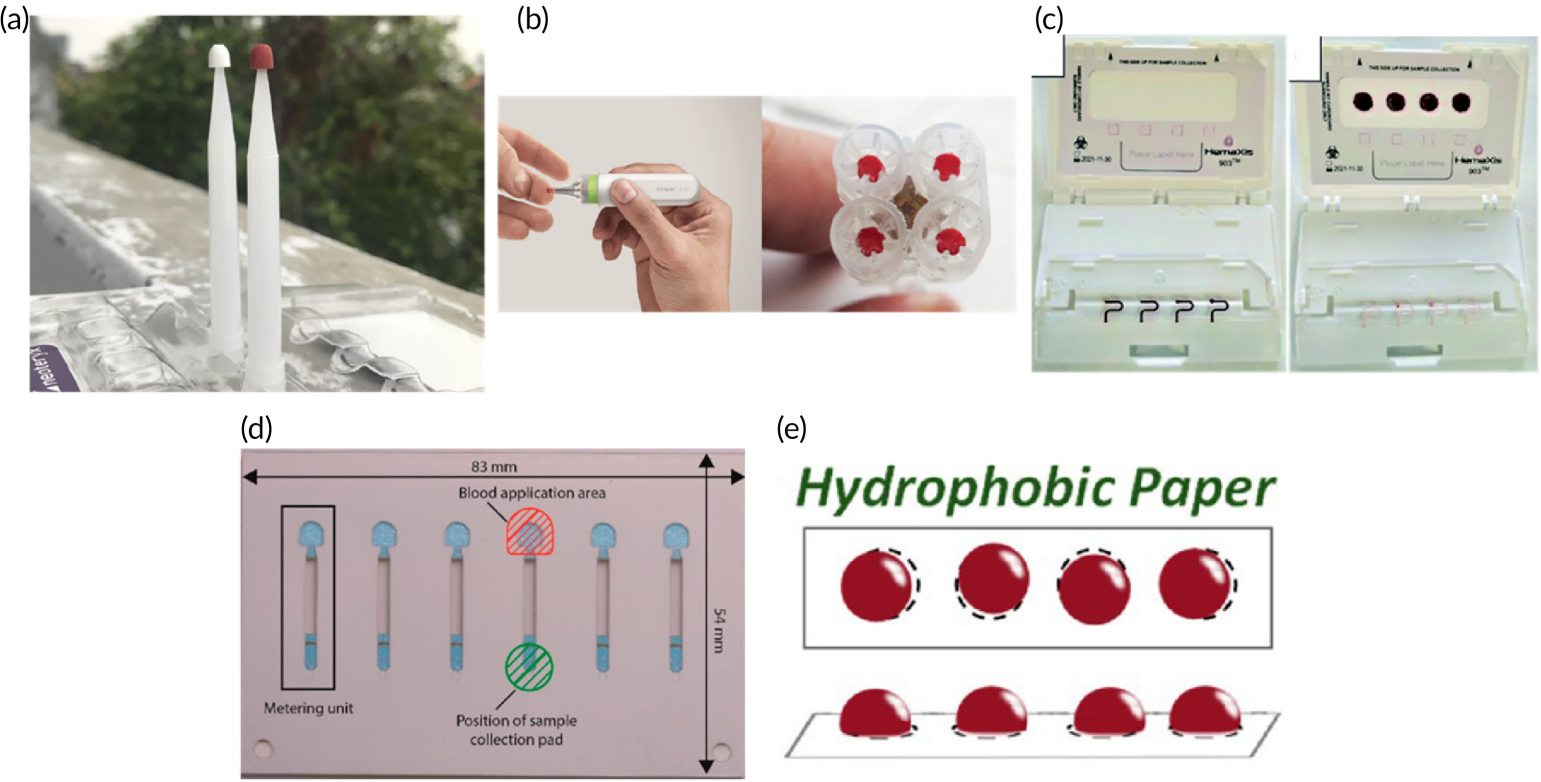
Alternative volumetric dried blood spot (DBS) technologies. These devices present novel approaches for minimizing the hematocrit effect. (a) Mitras volumetric absorptive microsampling device (VAMs) by Neoteryx restricts sample volume to the absorbent polymeric tip but can be affected by touching surfaces after collection or oversaturating (Dove Medical Press Limited, CC BY 3.0, Ref 66). (b) HemaPen by Trajan Scientific and Medical (Reprinted from Ref 49, Copyright 2019, with permission from Elsevier) and (c) HemaXis DB device by DBS System SA operate independent of the hematocrit by incorporating fixed‐volume capillary tubes, which dispense a discrete volume of blood onto filter paper (Republished with permission of Royal Society of Chemistry, from Ref 76; permission conveyed through Copyright Clearance Center, Inc.). (d) The Capitainer device by KTH protects against overfilling by inclusion of PVA valves that redirect excess sample prior to directing a discrete volume of sample to filter paper (Reprinted with permission from Ref 80, Copyright 2019 American Chemical Society). (e) In contrast, 3D blood spheroids utilize hydrophobic treated paper to eliminate chromatographic effects (Reprinted with permission from Ref 83, Copyright 2018 American Chemical Society).

In contrast, volumetric absorptive microsampler (VAMS) technology deviates from standard dried blood sampling and aims to provide simplified collection of blood by obviating additional tools for accurate volume application.^64^ This device comprises an absorbent polymeric tip sized to contain a discrete volume of blood (ca. 10 μl) attached to a plastic handle (Figure 6a).^65^, ^66^ The absorbent tip of the device is dipped into a drop of blood until it is fully saturated. Then, the pen is stored in a special case to ensure the tip does not contact any surfaces, which could alter the contained volume or result in contamination.^67^ Initial evaluation by gravimetric analysis yielded an average absorbed volume of 10.6 ± 0.4 μl blood across a hematocrit range of 20–65%. This result was supported by a previous study using radioactive ^14^C caffeine, which yielded a similar volume of 10.5 ± 0.1 μl.^68^ This device has been extensively evaluated for sampling accuracy in comparison to DBS technologies using a range of target analytes including: phosphatidylethanol,^69^ anthelmintic drug moxidectin,^70^ tacrolimus trough,^71^ and potassium.^72^ The devices described above each demonstrate improved volumetric sampling compared to traditional DBS cards through physically limiting the hydrophilic void available for blood collection. In contrast, the following devices employ fixed‐volume capillary tubes or integrated microfluidic components to ensure volumetric sample application with simple user interface.

The HemaPEN comprises four end‐to‐end EDTA‐coated microcapillaries (2.74 μl each) and four identical paper disks (3.5 mm diameter) within a pen‐like holder containing desiccant (Figure 6b).^73^ Accurate sampling is facilitated by the inclusion of fixed‐volume capillaries. Housing all four capillaries within a single device simplifies operation and minimizes user error. Specifically, undersampling and oversampling experiments demonstrated little variation when sampling time deviated from the recommended 10 s. This device demonstrated only a limited hematocrit dependence for the quantitation of caffeine by UPLC–MS/MS (7% difference across 20–50% hematocrit).^74^

Two book‐style devices also integrate fixed‐volume capillary channels to apply discrete volumes of blood to traditional DBS filter papers. The Hemaxis DB10 device comprises four fixed‐volume capillary channels (e.g., 5 or 10 μl) and removable filter paper to generate blood spots without needing a pipette (Figure 6c).^75^, ^76^ Blood is collected directly from a finger stick into each individual capillary. Closing the lid of the device brings the filter paper into contact with the capillaries to produce four replicate blood spots. Completely filling the capillaries and removing the entire blood spot ensures volumetric sampling independent of the hematocrit effect. Comparable recovery of phentermine was reported using the microfluidic‐based sampling device and blood spots generated with a volumetric pipette.^77^ In contrast, the Capitainer‐B device offers a single inlet port, which diverts a finger‐stick sample to a capillary microchannel designed to hold approximately 13.5 μl of blood independent of the input volume.^78^, ^79^ Volume metering is controlled in this device by thin dissolvable PVA films covering the waste pad (to absorb excess sample) and the precut sample pad (Figure 6d). This format was designed to simplify sampling by direct addition of unmetered blood drops. Gravimetric analysis yielded an average output sample volume of 14.25 ± 0.43 μl in the sample pad (26–62% hematocrit).^80^ Applying various sample input volumes (25–50 μl) resulted in ≤6.2% CV. Additionally, the quantitation of caffeine using the Capitainer‐B device yielded ±13.5% error in comparison to a liquid reference sample by LC–MS/MS. These results support that the Capitainer‐B device operates independently of both the hematocrit and applied blood volume.

The ADX Test Card by Accel Diagnostics comprises a microfluidic network to collect, distribute, and analyze blood.^81^ This technology leverages the aggregation of magnetic beads in response to the presence of biomolecules to produce quantitative results without an external reader.^82^ The size and quantity of aggregates captured by an internal magnet changes the flow of blood inside the microfluidic chip and is proportional to the concentration of target. The sample input volume is approximately 20–80 μl and distance‐based results are available after 10–15 min. Alternatively, three‐dimensional (3D) blood spheroids have been described using hydrophobic filter papers to overcome some of the limitations associated with traditional DBS cards.^83^ Whatman 1 filter paper was functionalized with trichloro(3,3,3‐trifluoropropyl)silane to produce a hydrophobic surface for applying blood samples (Figure 6e). In this format, the blood droplets dry on top of the paper rather than being absorbed and distributed throughout the porous matrix. Decreasing the interaction of blood in paper simplifies complete recovery of the sample (i.e., no lengthy extraction protocols), eliminates chromatographic effects due to uneven sample spreading, and stabilizes hydrolytically labile chemicals (i.e., only outer layer is exposed to air during drying) such as cocaine and diazepam.

Combining the accuracy of volumetric sample collection with enhanced analyte stability under ambient conditions increases the feasibility of self‐sampling low volumes of blood for expanding access to health diagnostics. However, the complex matrix of whole blood—specifically, the presence of blood cells—imposes a significant limitation on testing capabilities. In fact, many clinically established reference ranges for critical health evaluations are reported for samples of cell‐free plasma or serum rather than whole blood. For example, HIV viral load testing requires cell‐free plasma due to integrated proviral DNA found within infected T‐cells.^84^, ^85^ Similarly, RBCs contain high amounts of analytes also found in plasma (e.g., folate, iron, potassium), which can be released through disruption of RBC membranes and artificially elevate measured concentrations and lead to inaccurate test results for nutritional deficiencies, anemia, or even kidney disease, respectively.^86^ Ultimately, the presence of blood cells can introduce undesirable analytical interferents and obscure colorimetric read‐out techniques, which are common for many rapid point‐of‐care tests. For these reasons, extending the tools developed for sampling whole blood to include the separation of plasma could greatly increase testing capabilities and improve sample collection at the point‐of‐care.

## 3 SEPARATION OF CELLULAR AND LIQUID COMPONENTS OF WHOLE BLOOD AT THE POINT‐OF‐CARE

Methods for separating the cellular and liquid components of liquid blood can be categorized as either active (i.e., requires external instrumentation or sustained mechanical input by the user) or passive (i.e., only requires sample collection without additional user input or external instrumentation) with each approach presenting unique advantages and opportunities for microsampling at the point‐of‐care. The gold standard method for actively separating blood in a laboratory setting is centrifugation. Large volumes (e.g., ≤10 ml per tube) of whole blood are routinely processed in batches yielding nearly quantitative recovery of cell‐free plasma or serum with minimal risk of hemolysis under moderate centrifugal forces (ca. 800 g for 10 min). While centrifugation is highly effective and reproducible, the requirements present several challenges for use at the point‐of‐care including reliable electricity, bulky size, and cost (e.g., ≥$500).^87^ In contrast to active centrifugation, blood cells will passively sediment under gravitational force by simply leaving blood on the benchtop—eliminating the need for electricity. However, natural sedimentation such as this occurs on the order of multiple hours (>4 h) with poor yield (approximately 27% separation).^88^ Additionally, liquid plasma requires cold‐chain storage to maintain analyte stability, which further complicates sampling in rural or resource‐limited settings.

Emerging technologies utilize myriad techniques to address the current challenges associated with processing blood away from centralized laboratories and clinics including: (i) hand‐powered devices that output liquid plasma, (ii) passive separation devices that store dried plasma, (iii) passive separation devices that directly integrate with diagnostic tests, and (iv) membrane‐free passive separation technologies. Each approach presented here aims to facilitate field collection with emphasis on producing high quality analytical samples to expand access to critical health assessments. Distinct classes of devices are organized according to their design features and functionality. For example, devices that can separate plasma from cells, thus producing a liquid plasma sample ready for immediate use, can be identified by the term “liquid plasma output.” Alternatively, devices that can separate plasma from cells, and subsequently store plasma in a dried format, can be identified by the term “dried plasma output.” Standardizing performance metrics allows for direct comparison between different separation methods. Herein, we define separation efficiency as the ratio of recovered plasma volume to theoretical plasma volume (Equation (1)). For example: an input sample volume of 100 μl at a hematocrit of 45% would yield a theoretical plasma volume of 55 μl. If a hematocrit value is not reported, then a hematocrit value of 50% will be assumed.

(1) $$\text{Theoretical plasma volume}=\text{input sample volume}*\left(1-\text{hematocrit}\right)$$

### 3.1 Active, hand‐powered plasma separation devices for liquid plasma output

**FIGURE 7:**
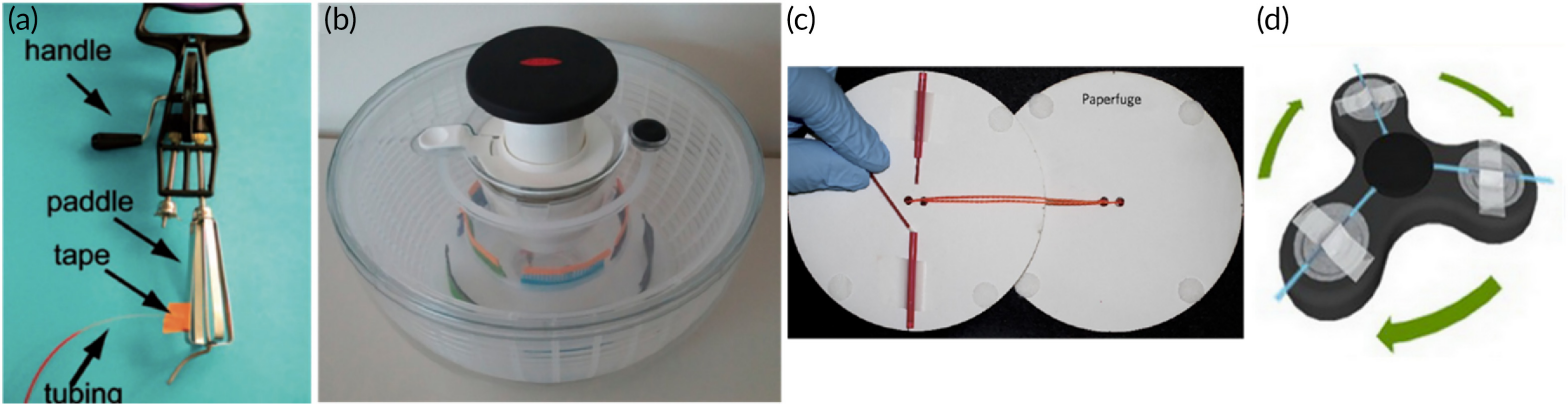
Hand‐powered centrifuges. (a) Eggbeater adapted with tubing containing blood (Reprinted with permission from Ref 88). (b) Salad spinner outfitted with a series of combs to hold microcapillary tubes containing blood (Reprinted with permission from Ref 89). (c) Low‐cost paperfuge with capillary tubes contained within straw segments (Reprinted by permission from Springer Nature: Ref 90, Copyright 2017.). (d) Unmodified fidget‐spinner toy with individually sealed capillary tubes on each arm (Reprinted with permission from Ref 91. Copyright 2019 American Chemical Society). Each device requires considerable energy and time input by the user.

Hand‐powered equipment represent an active method of separation and maintain the advantage of an applied centrifugal force for achieving fast separation, while obviating the need for electricity. These technologies generally comprise inexpensive household items that—with simple modifications—can process blood samples and yield liquid plasma (Table 2). In 2008, Wong et al. fitted a standard eggbeater with polyethylene tubing attached to a single paddle to process 100 μl samples of whole blood (Figure 7a).^88^ Samples were loaded into the tubing with a rubber bulb and each end was sealed using a flame and pliers. Rotating the handle at 200 revolutions per minute (rpm) for 8 min yielded maximum separation efficiency at 58% and produced a pellet of cells at the distal end of the tube. Liquid plasma samples were recovered by cutting the tubing at the interface with scissors or a knife. Approximately 50,000 cells ml^−1^ remained in the plasma sample obtained from the eggbeater. In contrast to a standard centrifuged sample of blood, the interface between cells and liquid plasma achieved by the eggbeater was more diffuse, resulting in a high number of remaining cells in the sample. Longer separation time or a higher rpm may improve separation, but diagnostic utility was unaffected for the detection of cholesterol as demonstrated by Wong et al.

**TABLE 2:** Plasma separation using hand‐powered centrifuges

Technology	Input volume (μl)	Output volume (μl)	Separation time (min)	Plasma quality	Reference
Eggbeater	100	58	10	>99% cell free	^88^
Salad spinner	14	NR	10	NR	^89^
Paperfuge	20	8	1.5	>99% cell free	^90^
Fidget‐spinner	10	3	7	>99% cell free	^91^

Similarly, a salad spinner was modified by Brown et al. in 2011 to accommodate up to 30 samples of whole blood for the measurement of hematocrit (Figure 7b).^89^ Low volumes of blood (14 μl) were contained in hematocrit capillary tubes and sealed at one end using putty sealant (opposite end of the tube remained unsealed). Two rows of fine‐toothed combs were affixed to the inside of the salad spinner to hold the capillary tubes at a fixed angle of 70°. Maximum separation efficiency was achieved following 10 min of pumping the salad spinner at a rate of 120 beats per minute (aided by a metronome) representing 602 rpm (average value for nine operators). Hematocrit values obtained by the hand‐operated salad spinner consistently yielded a 14% increase in packed cell volume compared to an electric‐powered centrifuge (ZIPocrit). In this study, liquid plasma samples were not recovered for volume estimation or purity; however, the microhematocrit tubes could be separated at the interface using scissors or a sharp blade to utilize the liquid plasma for a diagnostic assay.

Recently, two hand‐powered devices inspired by children's toys were reported for the separation of plasma. First, a modified paper‐based version of a toy whirligig—termed paperfuge (paper‐centrifuge)—was developed for measuring hematocrit of 20 μl aliquots of blood in less than 90 s (Figure 7c).^90^ Briefly, the device comprises two paper disks, wooden dowels (handles), string, capillary tubes, and straws (capillary tube holders). Simply twisting the strings by rotating the handles in opposite directions primes the device. While gently pulling on the strings in a rhythmic fashion facilitates rotation of the paper disks and subsequent separation of plasma from blood cells. Optimal separation efficiency of 40% was achieved with an output volume of 8 μl cell‐free plasma (>99% cell‐free confirmed by microscopy). Hematocrit values measured from samples prepared with the paperfuge were comparable to reference values obtained via centrifugation. The second device features a commercially available fidget‐spinner capable of separating three aliquots of blood (10 μl each) in under 7 min (Figure 7d).^91^ Blood was added to polyethylene tubing (50 mm long) before sealing both ends using a flame and pliers. Each tube was then taped to an individual arm of the toy perpendicular to the axis of rotation. Continuous rotation at an average speed of 1200 rpm yielded a separation efficiency of 30% producing approximately 3 μl of ≥99% cell‐free plasma (evaluated by microscopy). Diagnostic utility of the recovered plasma sample was demonstrated by quantitation of the HIV‐1 p24 capsid protein using a paper‐based enzyme‐linked immunosorbent assay (p‐ELISA) with excellent recovery (89–99%).

These hand‐powered devices present viable options for actively separating low volumes of blood to produce low volumes of cell‐free liquid plasma without electricity or bulky equipment. The quality of plasma obtained using these devices was comparable to plasma samples obtained via centrifugation and offers excellent quantitation potential when paired with a pipette or fixed‐volume capillary. While the salad spinner can accommodate up to 30 samples at a time, the eggbeater, paperfuge, and fidget‐spinner each represent low throughput options. Additionally, the active input required by the user presents two problems: (i) high risk of exposure if the crimped tubing or putty sealant fails and (ii) high demand of time and energy from dedicated users. In contrast, passive separation techniques aim to minimize user‐steps and hands‐on time without the aid of electrically powered equipment.

### 3.2 Passive plasma separation devices for liquid plasma output

Porous materials comprise the basic approach for passive separation to effectively filter cells while providing a source of capillarity for transporting plasma to a collection medium (such as a capillary tube or absorbent pad). Plasma separation membranes (PSMs) are readily available in a variety of materials including glass fiber (e.g., Whatman LF1), asymmetric polysulfone (e.g., Pall Vivid), and blended natural and synthetic fibers (e.g., Ahlstrom CytoSep). Each material presents unique advantages with respect to loading capacity, void volume, separation time, orientation (i.e., lateral vs. vertical separation), and final plasma purity as defined by the manufacturer. While these characteristics provide excellent guidelines for use, opportunities exist for reimagining their application to improve performance metrics related to separation efficiency. The following devices deviate from the standard application of commercial PSM to dramatically increase separation efficiency by (i) reorienting the PSM, (ii) taking advantage of natural sedimentation by gravity, and (iii) including a prefilter column treated with reagents to capture RBCs (Table 3). Each device passively separates plasma and yields cell‐free liquid plasma, which can be recovered using volumetric tools such as a capillary tube or micropipette.

**TABLE 3:** Passive separation techniques for plasma processing and liquid plasma output

Separation mechanism	Input volume (μl)	Output volume (μl)	Separation efficiency	Separation time (min)	Plasma quality	Reference
Size exclusion	20 (diluted)	10	NA	0.18	<5 mg/dl hemoglobin	^92^
Size exclusion/sedimentation	1800	275 ± 34	30%	7	3.5 ± 1.2 mg/dl hemoglobin	^94^
Size exclusion/sedimentation	200	65 ± 22	65%	10	NA	^96^
Size exclusion/immunocapture	400	131.8 ± 3.4	94%	5	>99% cell free	^97^

**FIGURE 8:**
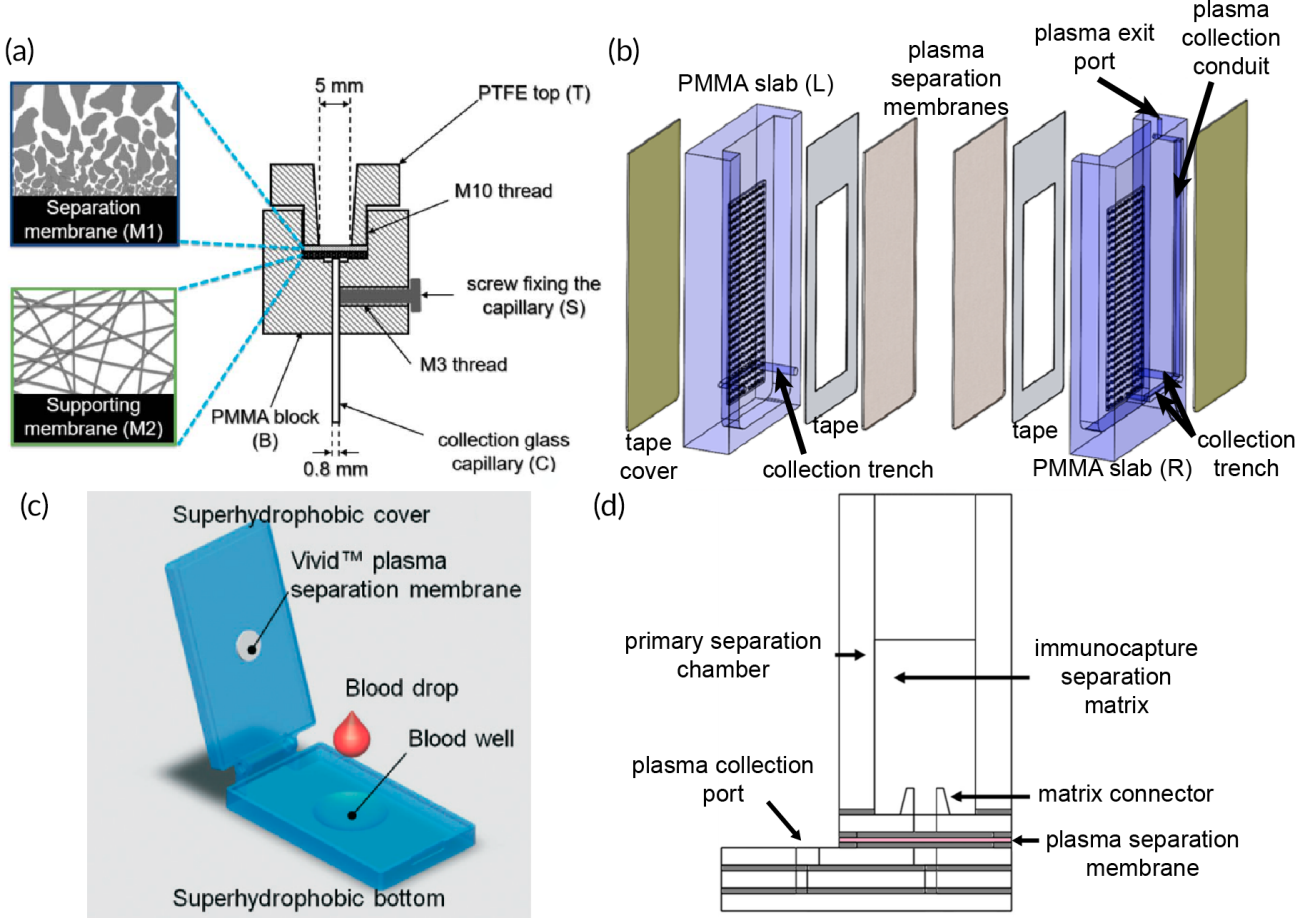
Passive separation techniques and liquid plasma output. Each device incorporates at least one layer of plasma separation membrane for filtering cells by size exclusion. The first device (a) collects diluted liquid plasma directly into a capillary tube (Reprinted from Ref 92, Copyright 2018, with permission from Elsevier). In contrast, the following devices require a pipette to provide a source of vacuum for recovering plasma. Devices (b) (Reprinted with permission from Ref 94, Copyright 2013 American Chemical Society) and (c) (Reprinted with permission from Ref 96) operate by size exclusion and sedimentation. While device (d) operates by size exclusion and immunocapture (Reprinted with permission from Ref 97).

Untreated, unmodified PSM oriented for vertical flow provides the baseline for performance.^92^ In this example, the PSM is a hydrophilic, asymmetrical polysulfone (Pall Vivid GR) material with a reported loading capacity of 40–50 μl blood cm^−2^.^93^ Durc et al. fabricated a device comprising a sample addition reservoir and polymethylmethacrylate (PMMA) scaffold to suspend the separation materials above a 10 μl capillary collection tube (Figure 8a). The membrane was cut to 8 mm diameter representing an area of 0.5 cm^2^ and an input volume of 20–25 μl whole blood. Directly beneath the PSM was a supporting material (nonwoven wiper, CLEANTEX), which provided an additional source of capillarity. Initially, Durc et al. applied 20 μl of undiluted whole blood yielding 1–10 μl of plasma. Increasing the volume of blood to 40 μl exceeded the loading capacity of the material, which clogged the pores of the membrane and stopped flow yielding no plasma. In order to maintain the relatively small footprint of the device for use at the point‐of‐care, Durc et al. diluted samples of whole blood 1:5 in an isotonic solution containing 7% [tris(hydroymethyl)methylamino] propanesulfonic acid to enhance agglutination of RBCs. Diluting the blood lessened the cellular burden on the PSM, improved reproducibility, and maintained a fast separation time (10 s) without causing hemolysis. While this approach and specific dilution formulation were successful for the quantitation of oxalate, formate, and glycolate in the presented device, it is usually preferred to use undiluted samples especially for analytes that exist in low concentrations (e.g., pg ml^−1^ quantities). However, this is an excellent demonstration of the rigidity of the reported loading capacities of PSM and presents opportunities for improving separation.

Orienting Vivid PSM horizontally facilitates vertical separation for even distribution of blood over the entire area of the membrane. Liu et al. reported a plasma separator using Vivid PSM positioned vertically to take advantage of natural sedimentation in addition to size exclusion.^94^ In this format, two rectangular pieces of Vivid grade GR (15 mm × 38 mm) were attached to opposite sides of a PMMA scaffold machined with a micropillar array and collection trench (Figure 8b). The total area of PSM in contact with blood during separation was approximately 4 cm^2^, which represents an input volume of roughly 200 μl whole blood. However, due to the unique orientation of the separation materials and sedimentation‐aided approach, Liu et al. were able to load the device with 1.8 ml of undiluted whole blood and recovered 275 ± 34 μl of plasma within 7 min. Orienting the membranes in this way also minimized shear forces experienced by the RBCs, resulting in negligible hemolysis at 3.5 ± 1.2 mg dl^−1^ hemoglobin, which fell below the reported threshold of interference for most assays (>5.0 mg dl^−1^).^95^ Liquid plasma was recovered from the collection trench via a pipette and was immediately applied to a point‐of‐care nucleic acid test for HIV viral load monitoring. Preliminary results indicated promising recovery of 81.5 ± 12.1% for viral loads as low as 350 copies ml^−1^. While this device boasts an impressive volume of recovered plasma (i.e., hundreds of microliters), the massive input volume results in a separation efficiency of approximately 30%, which is comparable to the baseline performance of PSM oriented horizontally.

Another example of reorienting Vivid PSM for enhanced separation efficiency was reported by Liu et al. with a high‐efficiency superhydrophobic plasma separator.^96^ The clamshell‐style device comprises a bottom slab with sample addition well and a hinged cover, which houses a Vivid GR PSM and plasma extraction port (Figure 8c). A superhydrophobic coating was applied to both the bottom well and cover to encourage the blood to interact with the PSM positioned above the sample. Situating the separation material and collection port above the blood sample benefits from natural sedimentation due to gravity during the separation and wicking process. Upon closing the lid, blood contacts the PSM and wicks into the pores of the PSM. Meanwhile, the cellular components of blood settle to the bottom of the sample well and cannot clog the PSM. Recovery of plasma is facilitated by a pipette to provide a vacuum source following 10 min of separation. In this configuration, the Liu et al. were able to achieve 65% separation efficiency with an input volume of 200 μl and an average extraction volume of 65 ± 22 μl. That is nearly double the expected extraction efficiency of using PSM in the traditional orientation (approximately 30%). However, the high standard deviation reported for average extraction volume (a CV of roughly 33%) indicates poor reproducibility of the method.

Increasing the baseline separation efficiency and plasma yield has also been achieved by a combination of immunocapture and size exclusion using Vivid GR PSM.^97^ The primary separation chamber of this device contains multiple layers of acetate fibers coated in RBC agglutination reagent (anti‐RBC) to capture majority RBCs before the sample reaches the final separation membrane (Vivid GR PSM) to exclude remaining cells (Figure 8d). Incorporating the immunocapture module upstream of the PSM increased the loading capacity twofold. A sample input volume of 400 μl whole blood (65% hematocrit) and recovery of 131.8 ± 3.4 μl liquid plasma yielded a separation efficiency of 94% in 5 min. Additionally, evaluation of the final plasma sample collected with a pipette indicated >99% of cells were excluded.

Historically, passive separation methods using porous membranes suffer from physical adsorption of target analytes, large void volume, and subsequent low separation efficiencies (ca. 30%). However, coupling commercial PSM with unique orientations and complimentary strategies have surpassed expectation with up to 94% separation efficiency.^96^ Similarly, reported recoveries for myriad analytes (e.g., proteins, viral RNA, and genomic DNA) demonstrate the excellent diagnostic utility of liquid plasma produced by passive filtration for immediate use. For applications where immediate use is not feasible (e.g., field collection) or for broad population studies (e.g., proteomics), storing separated plasma with enhanced stability is paramount.

### 3.3 Passive plasma separation devices for dried plasma output in porous materials

Enhanced sample stability over time has successfully been demonstrated for samples of whole blood dried in a porous matrix such as cellulosic paper and silk fibroin.^98^ This same approach of dry storage can be extended to samples of cell‐free plasma as an alternative to liquid storage to minimize the thermal degradation of proteins in the absence of cold‐chain storage.^99^ This simple approach of passive separation paired with dried storage has the potential to greatly expand access to routine health analyses—such as plasma viral load testing—as demonstrated by a recent population study.^100^ The following section includes both commercially available technologies as well as academic demonstrations for plasma separation and storage within a single device (Table 4). The majority of approaches presented here utilize a form of commercially available PSM and collection pad comprising filter paper to produce a wide range of output plasma volumes with excellent purity (i.e., minimal hemolysis).

**TABLE 4:** Passive separation techniques for plasma processing and dried plasma output stored in porous matrix

Technology	Input volume (μl)	Output volume (μl)	Separation efficiency	Separation time (min)	Reference
Plasma separator	75	27.4 ± 2.1	55.3%	4.3 ± 0.8	^101^
Plasma separator	250	65.6 ± 3.9	53.8%	10	^102^
Noviplex Uno	25	2.5	20.0%	3	^103^
Volume‐defined DPS	40–80	11.6 ± 0.3	58.0%	6	^104^
Roche PSC	140	~40	57.0%	5	^106^
Q2 device	10–50	4.6–14.7	55.0%	3	^107^
pDPS card	75	17.2	45.0%	5	^108^

Abbreviations: DPS, dried plasma spot; pDPS, patterned DPS; PSC, plasma separation card.

**FIGURE 9:**
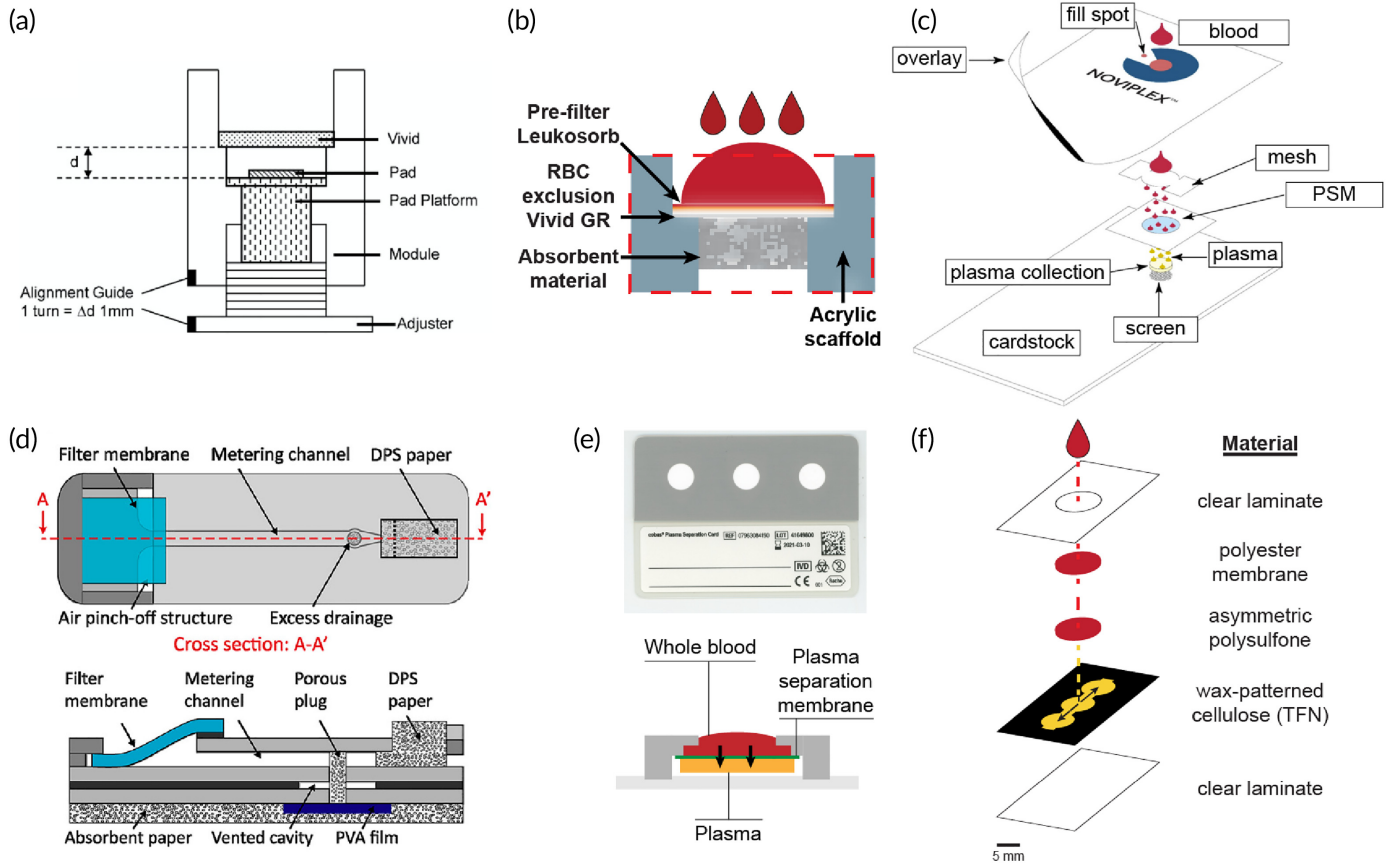
Passive separation techniques and dried plasma output stored in a porous matrix. Two vertically stacked devices enhanced separation efficiency by (a) treating the plasma separation membrane (PSM) layer (Reprinted from Ref 101, Copyright 2011, with permission from Elsevier) and (b) inclusion of a prefilter material (Reprinted with permission from Ref 102, Copyright 2020 American Chemical Society). Plasma separation cards comprise a basic structure of PSM layered with filter paper. The (c) Noviplex UNO produces a single plasma sample in a precut disk (Reprinted with permission from Ref 103, Copyright 2013 American Chemical Society). (d) The volume‐defined dried plasma spot (DPS) meters plasma using a PVA valve to remove excess plasma before filling an absorbent paper strip (Reprinted with permission from Ref 104, Copyright 2019 American Chemical Society). (e) The Cobas PSC by Roche produces a single sample of plasma in a shield‐shaped fleece located beneath the PSM. (f) The patterned DPS (pDPS) card incorporates a prefilter material and filter paper impregnated with hydrophobic barriers to control plasma flow and distribution for improved purity (Ref 108).

The first example employs an assembly of machined polypropylene parts supporting a Vivid GF PSM and collection pad positioned on a movable platform controlled by a screw mechanism (Figure 9a).^101^ Raising the movable platform initiates separation by providing a source of capillarity. Similarly, lowering the platform terminates separation and minimizes hemolysis. Vivid grade GF PSM has a reported loading capacity of 20 μl blood cm^−2^ and estimated 60% separation efficiency according to the manufacturer (e.g., 6.6 μl from 20 μl of 45% hematocrit sample). However, Nahatiyan et al. treated the Vivid GF PSM with a blocking solution (0.1% BSA, 0.5% sucrose, 0.1% Tween−20) to increase hydrophilicity, which also allowed the sample input volume to exceed the recommended loading capacity. Subsequently, an input volume of approximately 75 μl was applied to a membrane area of 1.91 cm^2^ representing a loading capacity of nearly 40 μl blood cm^−2^. Next, Nahatiyan et al. screened 12 materials including various grades of glass microfiber (e.g., 111, 121, 142, 151 Ahlstrom; A/B, A/D, A/E Pall Corp.); polyester (e.g., Accuwik and Leukosorb B Pall Corp.); Whatman Fusion 5 membrane; and cellulose (e.g., Type III Pall Corp.; grade 903 Whatman). Separation time (3.5 ± 0.5 min) and collection volume (25.8 ± 0.7 μl) were relatively constant with respect to the different collection pad materials. However, one collection pad (glass fiber A/D Pall Corp.) was ultimately selected because it displayed the lowest background signal while maintaining excellent recovery of the analyte of interest (immunocomplexed HIV p24 antigen). Final characterization yielded an average recovered volume of 27.4 ± 2.1 μl in 4.3 ± 0.8 min across a hematocrit range of 21–48%, representing an average separation efficiency of 55.3%.

Similarly, a construct of unmodified porous materials was described for processing larger volumes of blood (ca. 250 μl) to produce a high volume of cell‐free plasma demonstrating a comparable separation efficiency (Figure 9b).^102^ In contrast to the previous method, this approach benefits from the use of unmodified separation and collection materials to maintain compatibility with downstream assays. In this format, a Vivid GR PSM (1.9 cm diameter) was supplemented with a prefilter material (Leukosorb) and absorbent pad (ShamWow) to effectively increase the loading capacity twofold compared to manufacturer recommendations. The Leukosorb prefilter served to remove the majority of RBCs and WBCs by size exclusion and electrostatic interactions, while the Vivid GR PSM excluded remaining RBCs. The ShamWow provided excellent capillarity for recovery of liquid plasma and can either be (i) dried and stored for future analysis or (ii) “stamped” onto an LFT for immediate use. Maintaining relatively small membrane areas minimized the void volume to produce a high yield of liquid plasma (average 61.7 ± 2.1 μl) across the physiological range of hematocrits (20–60%). A maximum separation efficiency of 53.8% was achieved for a sample input of 250 μl undiluted whole blood (30% hematocrit) in 10 min. Purity of the recovered plasma was evaluated by quantitation of hemoglobin and was reported as either minimal (≤5%) or undetectable (≤1%). Similarly, recoveries of two distinct classes of biomolecules (e.g., proteins and viral RNA) indicated the diagnostic utility of plasma was unchanged compared to plasma obtained via centrifugation.

Plasma separation cards (PSCs) provide in‐line passive separation and storage of dried plasma for enhanced analyte stability at ambient conditions. PSCs present a similar basic structure between manufacturers comprising a single layer of PSM coupled with a porous collection pad located directly beneath the PSM. Once the PSCs have dried completely, the user peels the device apart to reveal the collection material and performs subsequent diagnostic or clinical tests. Beyond this basic structure, each card also incorporates unique features such as (i) a control spot for filling, (ii) sample splitting layer, and (iii) volume‐restricted collection pad. Additionally, sample input and output volumes vary widely depending on the manufacturer (Table 4). The Noviplex Uno PSC by Novilytic offers a low volume option (25 μl input) with a rapid drying time of only 15 min for a sample containing 2.5 μl of plasma (Figure 9c).^103^ A spreading layer positioned above the PSM serves two functions: (i) ensures even distribution of whole blood over the area of the PSM and (ii) indicates sufficient sample loading by directing blood to a control spot viewed from the top of the card. Similarly, the second‐generation card by Novilytic—Noviplex Duo—operates identically but accepts a larger input volume (≥60 μl) and splits the plasma into two distinct collection pads for a total recovery of 6.4 μl (3.2 μl per collection pad). These cards produce low volumes of plasma, which may preclude them from being used for analytes in low abundance where higher volumes are necessary. In contrast, a microfluidic device comprising a single layer of PSM (IPOC “SGR”), hydrophilic metering channel, water‐soluble poly(vinyl alcohol) film, and absorbent paper matrix (Ahlstrom grade 222) successfully generates volume‐defined dried plasma spots (Figure 9d).^104^ A critical feature of this device is the use of an air pinch‐off structure, which controls air inflow dependent on a unique geometrical constriction channel below the PSM.^105^ Excess plasma is first removed from the metering channel via a porous paper plug and vented cavity. Then, the PVA film is dissolved to allow absorption of plasma into a blotting paper layer below. Gravimetric analysis yielded a volume of 11.6 ± 0.3 μl plasma independent of the input volume of blood (40–80 μl whole blood), representing a separation efficiency of approximately 58%. Currently, the Roche Cobas PSC produces the highest volume of plasma obtainable using a commercial card and is sold as a consumable for pairing with a Roche clinical analyzer (Cobas 6800/8800 system) for HIV‐1 testing.^106^ The Roche Cobas PSC comprises a PSM in direct conformal contact with a shield‐shaped fleece treated with RNA‐stabilizing reagents (proprietary formulation) supported by a base cardstock material for patient identification (Figure 9e). This card requires a higher input volume of 140 μl whole blood and yields approximately 40 μl plasma for a separation efficiency of nearly 57%. Higher plasma volumes are needed for the intended application of this card (i.e., HIV‐1 testing with the Cobas analyzer).

In contrast to the single layer PSM PSCs above, the Q2 book‐style device comprises a series of stacked membranes and porous materials contained within a folded cardstock “book” to generate uniform plasma spots.^107^ Two PSMs (iPOC^DX^ grades X and S/G) were combined to filter cells and minimize hemolysis. The first layer of PSM (grade X, 5 mm diameter) was physically punctured to improve sample flow to the second PSM below (grade S/G, 7 mm diameter). A layer of polyester (Ahlstrom Hollytex 3256) served to hold the S/G membranes in place and prevent liquid plasma from spilling over the edge onto the collection pad thus improving spot uniformity. A series of cardstock disks were positioned below the cellulose collection layer (Ahlstrom 601) to ensure conformal contact with the separation materials when the book‐style device was closed. In this configuration, an input sample volume of 20 μl whole blood (45% hematocrit) yielded approximately 6 μl of plasma in 4 min representing a separation efficiency of 55%. Increased separation efficiency using the book‐style device is attributed to the thinner grade X PSM (160–200 μm), which is approximately half the thickness of grades S/G and Pall Vivid GR (300 and 330 μm, respectively). The thinner membrane effectively decreases the void volume of the separation device. A comparable technology—the patterned dried plasma spot (pDPS) card—layers two separation membranes on top of a standard DBS cardstock with defined lateral channels and plasma collection zones (Figure 9f).^108^ Hydrophobic wax barriers direct the flow of plasma away from the separation materials to yield two identical sample collection zones designed to be removed using a standard 6‐mm punch. Spatially separating the plasma samples from the separation materials ensures high purity by minimizing hemoglobin leakage. pDPS cards accommodate a sample input volume of 75 μl and total output plasma volume of 17.2 μl plasma (8.6 μl per 6‐mm punch) yielding a separation efficiency of approximately 45%. The above devices utilize vertically stacked materials for passive generation and storage of plasma. A similar method of separation has also been achieved using lateral flow in several configurations.

### 3.4 Passive, lateral flow plasma separation devices for dried plasma output

**FIGURE 10:**
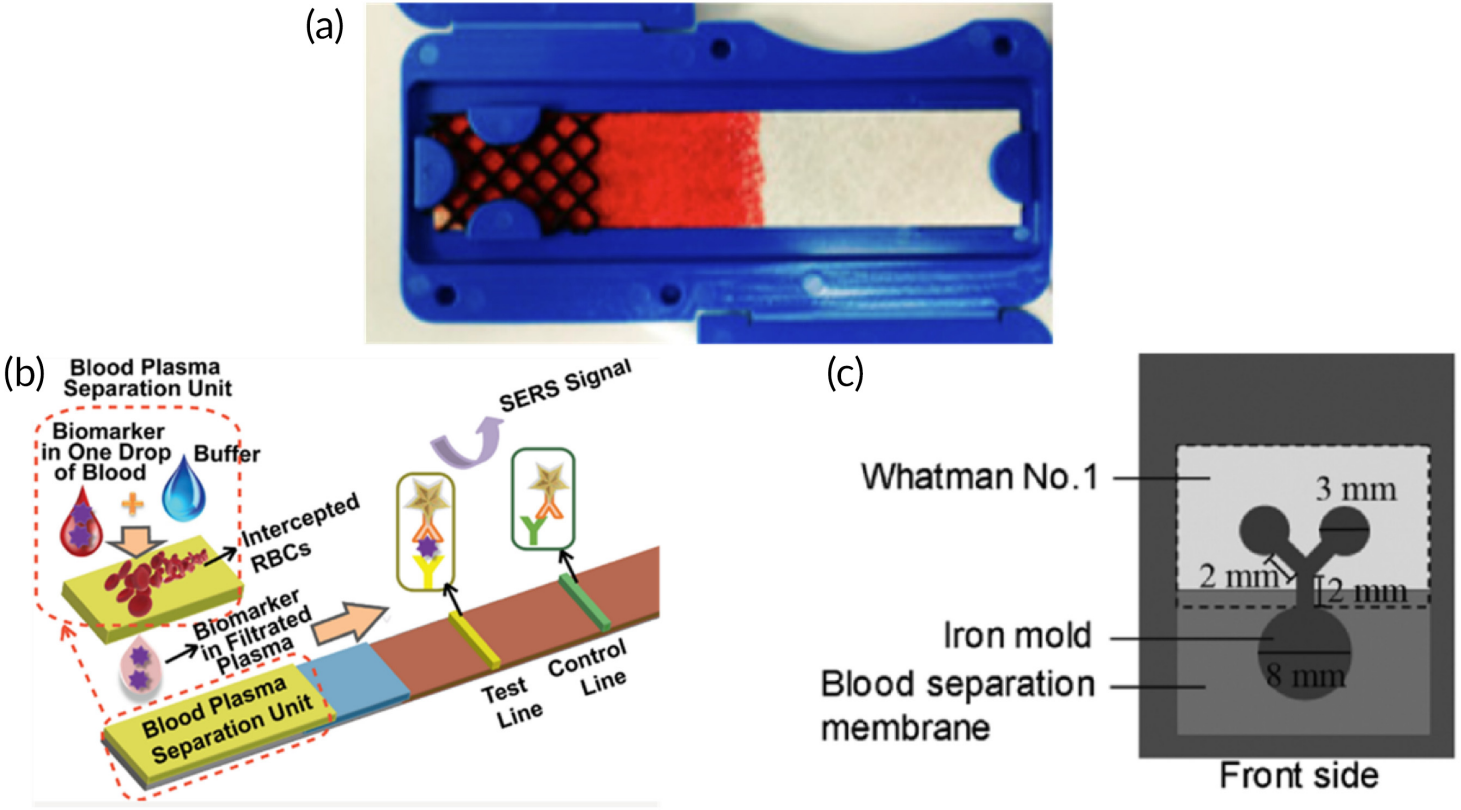
Lateral flow separation devices. (a) The blood collection device comprises a spreading mesh and rectangular strip of glass fiber to produce a gradient of plasma (Reprinted with permission from Ref 110). A lateral flow test with upstream plasma separation unit (b) comprising three layers of stacked plasma separation membrane (PSM) with unique chemical treatments for increased hydrophilicity (Reprinted with permission from Ref 116, Copyright 2021 American Chemical Society). (c) Microfluidic paper analytical device (μPAD) comprising overlapped glass fiber separation membrane and filter paper patterned with hydrophobic barriers for detection of plasma proteins (Reprinted with permission from Ref 121, Copyright 2012 American Chemical Society).

LFTs benefit from highly automated roll‐to‐roll manufacturing methods, which are well established and present a clear path to large‐scale production.^109^ However, blood‐based LFTs can suffer from high background signal interference due to the intrinsic red color of hemoglobin. To remove this coloration, blood samples are routinely processed off‐chip which incurs additional user‐steps and presents a considerable barrier for adoption in the field or at the point‐of‐care. Integrating plasma separation directly into LFTs while maintaining automated manufacturing methods could expand testing capabilities at the point‐of‐care. A lateral flow blood collection device (BCD) was fabricated to separate plasma while maintaining protein integrity at ambient storage conditions for proteomic applications.^110^ The device comprises a spreading mesh, rectangular strip (85 mm × 17 mm) of glass fiber membrane (Whatman LF1), and desiccant housed within a plastic casing (Figure 10a). The minimum sample input volume required is 250 μl to adequately fill the entire membrane strip indicated by the sample reaching the distal end opposite of sample addition. The spreading mesh ensures even distribution of the blood sample across the width of the membrane. Desiccant is positioned directly above the sample addition end of the membrane to reduce dry time and subsequent hemolysis. As a result, spectral hemoglobin determined by MALDI‐ToF was generally low (0–6%) for all devices tested. However, there was evidence of increasing hemoglobin contamination across the membrane for punches obtained in proximity to the sample addition zone. This was attributed to RBC rupture during the drying process and lateral wicking across the membrane. Similarly, a significant protein gradient was observed (*p* = 0.0045) across the length of the membrane containing the separated plasma. Kaiser et al. noted that underfilling the device resulted in excessive hemolysis due to strong capillarity. Commercially available devices such as the ADx100 card by Advance Dx and the HemaSpot SE by Spot on Sciences also utilize lateral separation membranes with varying form factors and input volumes. The ADx100 card comprises a simple rectangular strip of lateral separation membrane affixed to a piece of cardstock and accommodates up to four drops of blood.^111^ Similarly, the HemaSpot SE accommodates two to six drops of blood and produces cell‐free plasma along a spiral membrane within a plastic housing.^112^ The resultant sample gradient contains majority cells in the center and pure plasma near the tail end. Samples obtained via punching along the spiral yielded varying protein concentrations with the highest concentration resulting at the end of the spiral. These examples clearly demonstrate the consequent chromatographic effects of separating plasma by lateral flow.

Chlorine was used as a normalizing analyte for lipid measurements using the Velvet device by Weavr Health to correct the analyte gradient across a lateral separation membrane.^113^ This device is structurally similar to the BCD described above, comprising a lateral separation membrane contained within a plastic cassette. However, this device also integrates three fixed‐volume capillaries for accurate sample addition of 180 μl blood. Chlorine was selected as the normalization analyte due to its low variation between samples (1.3%) and excellent stability over 28 days at ambient conditions. Subsequent punch‐to‐punch variations across the separation membrane averaged 18.7% and systemic under‐recovery was reported even with chlorine normalization. While quantitation of target analytes using lateral flow separation can prove highly variable, these devices can detect the presence or absence of myriad analytes to inform diagnosis of various disease states.^114^, ^115^ Additionally, several examples utilize multiple PSMs stacked with partial overlap to form a hybrid of vertical and lateral flow to achieve semiquantitative results when paired with an external reader (e.g., cell phone).

Recently, two LFTs have been reported, which employ lateral flow plasma separation units positioned upstream of detection zones for quantitation of various proteins. First, a standard paper‐based lateral flow strip (i.e., conjugate pad, nitrocellulose membrane, absorption pad, backing membrane) was fabricated with three layers of surface‐modified PSM (Vivid GR 5.5 cm × 3 cm) and surface‐enhanced Raman scattering (SERS) probes (Figure 10b).^116^ A single layer of PSM was insufficient for removing majority RBCs from the sample prior to reaching the conjugate pad. Combining three or more layers of PSM in a cascading fashion (1.5 mm overlap between membranes) provided enhanced separation and sample purity. However, additional modifications to the PSM were necessary for desired flow dynamics in this device. Two layers of PSM (layers 1 and 3) were treated with sodium chloride to induce aggregation of RBCs. The second layer was treated with a surfactant (Tween 20) to increase hydrophilicity for improved plasma flow to the final layer of PSM. This combination of PSM layers yielded a separation efficiency of 30% after 20 min with a sample input volume of 30 μl blood. Plasma purity was sufficient for quantitation of carcinoembryonic antigen by SERS with a limit of detection of 1.0 ng/ml.

Similarly, the high‐yield passive erythrocyte removal (HYPER) device employs three different materials to facilitate separation of plasma from whole blood in conjunction with a multiplexed LFT for evaluating malnutrition by fluorescence.^117^ The sample processing portion of the device comprises a differentiation membrane to prevent biofouling and remove majority RBCs prior to reaching a Vivid GF PSM for final RBC exclusion. A calibration pad (Whatman Fusion 5; Cytiva) provided capillarity and stored separated plasma prior to initiating the LFT with buffer. The calibration pad contained a discrete volume of plasma informed by the reported capacity of 40 μl cm^−2^. This ensured accurate sampling between blood samples with different hematocrit values or if excess blood was applied to the device. However, it is contingent upon removal of the upper separation materials (differentiation pad and PSM) once the calibration pad is visually saturated with plasma. The HYPER device accommodated up to 60 μl of blood and yielded an average separation efficiency of 66% within 10 min. Extending separation time for a total of 20 min produced a higher separation efficiency of 81.6%. While sufficient plasma volume was produced to perform a multiplexed LFT, strip‐to‐strip variation for the multiplexed LFTs was high (>20% for each analyte). Lateral flow separation is not limited to LFTs, rather, this same approach has been extended to 3D microfluidic paper analytical devices (3D μPADs).

3D μPADs represent an attractive alternative to traditional LFTs.^118^ These devices integrate sample preparation and storage of necessary reagents for on‐chip detection or quantitation using simple methods of signal generation (e.g., colorimetric) to minimize the need for external readers and equipment.^119^, ^120^ A two‐layer device comprising glass fiber separation membrane (Whatman LF1, 1.7 cm × 2.5 cm) and cellulose chromatography paper (Whatman 1, 1.5 cm × 2.5 cm) were overlapped (1 mm) and joined by a Y‐junction to form two detection zones using hydrophobic barriers (Figure 10c).^121^ The sample input volume of blood scaled with the area of the device. Songjaroen et al. presented two device geometries: (i) 28 mm^2^ (input 8–11 μl blood) and (ii) 50 mm^2^ (15–22 μl blood). Plasma purity was assessed by microscopy using a separation membrane diameter of 8 mm. RBCs remained in the separation membrane and did not enter the Y‐junction for volumes ≤15 μl. However, if the sample was increased to 30 μl, RBCs entered the Y‐junction indicating leakage. Bromocresol green indicator was stored in the cellulose test zones for protein quantitation. There was no statistical evidence of differences in the means for quantitation of total protein (*p* = 0.437) and no visual interference of RBCs or hemoglobin in the test zones for sample input volumes in the recommended range. Further simplifying the fabrication scheme of this 3D μPAD by combining the separation and collection of plasma within a single layer of material using scalable methods could lower manufacturing barriers.

Recently, a single layer device fabricated from the same glass fiber separation membrane (Whatman LF1) was reported comprising the same geometry and design features as the device described above.^122^ In this example, the LF1 membrane was treated with a two‐step process: (i) first the membrane was coated with a fluorocarbon layer using pentafluoroethane plasma deposition to render the backside of the material hydrophobic followed by (ii) application of a mask and O_2_ plasma etching to imprint the working area of the device for separation and detection within the single layer of LF1. A sample input volume of 9–12 μl blood was applied to the device for the colorimetric detection of glucose and albumin. Fabrication methods presented here are amenable to roll‐to‐roll manufacturing and represent potential for large‐scale production.

Another challenge when fabricating devices with multiple layers of materials is achieving and maintaining conformal contact between layers, and between plasma separation membranes and absorbent materials in particular. Specifically, separation efficiency is greatly affected by the degree of contact between such materials. To address this, a 3D μPAD comprising Vivid GR PSM and Whatman 1 filter paper was fabricated to eliminate the potential gap between the two materials.^123^ In this approach, Park et al. printed a sample reservoir and detection zones directly onto the PSM and filter paper assembly by liquid photopolymerization using a digital light processing printer. This printing method eliminated multi‐step assembly processes for streamlined manufacturing. First, the PSM was protected from dissolution in organic solvent (photocurable polymer) using parylene C. Next, the PSM surface was treated with oxygen plasma and superimposed onto the filter paper. Finally, the sample reservoir and detection zones were defined using a 3D printer. The device accommodates a sample input volume of 100 μl of blood for the direct quantitation of glucose using colorimetric analysis. As demonstrated by the devices in this section, PSMs are among the most used materials for passively separating plasma from whole blood. While the benefits of these materials are well recognized, they are severely limited by (i) rigid loading capacities, (ii) are prone to degradation dependent on ambient conditions/storage, and (iii) can absorb a considerable volume of the desired plasma sample (i.e., “dead” or void volume). Maintaining the advantages of passive separation while minimizing the intrinsic limitations of commercial PSMs could improve microsampling of plasma.

### 3.5 Alternative methods for passive separation of plasma without PSM

**FIGURE 11:**
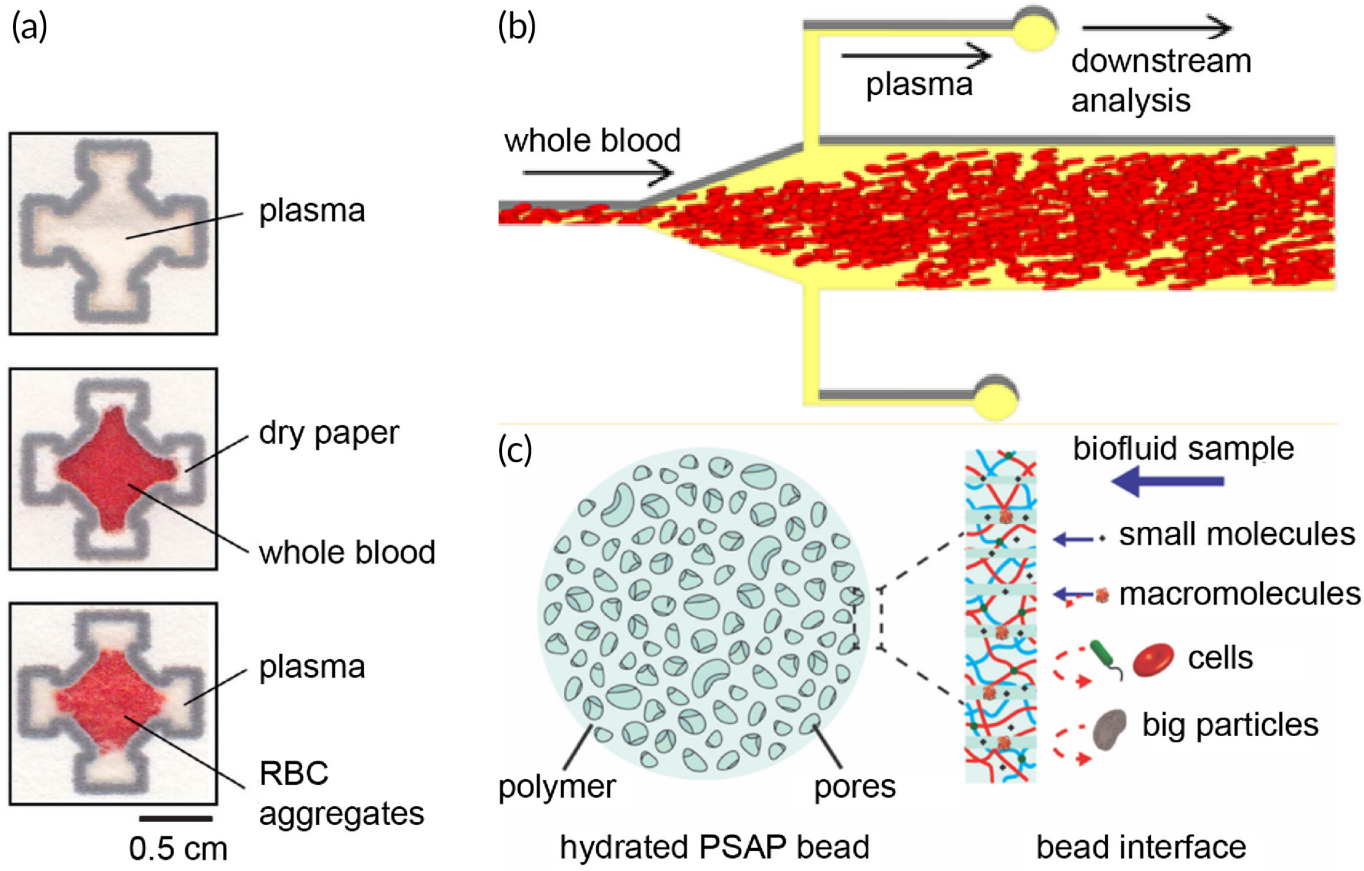
Plasma separation membrane (PSM)‐free separation technologies. (a) Agglutination‐based approach in a single layer of filter paper with four plasma output zones (Reprinted with permission from Ref 125). (b) A microfluidic device utilizing constriction‐expansion inertial cell sorting and liquid plasma collection (Reprinted with permission from Ref 132, Copyright 2016 American Chemical Society). (c) Porous superabsorbent polymer (PSAP) beads for separation and storage of plasma by size exclusion (Reprinted with permission from Ref 133).

Agglutination‐based separation techniques represent an attractive alternative to PSM alone. Proteins such as wheat germ agglutinin^124^ and blood‐typing antibodies^125^, ^126^ can be used to agglutinate RBCs. In 2012, Yang et al. reported a single layer μPAD comprising Whatman 1 chromatography paper functionalized with A/B antibodies to yield a dried plasma output.^125^ Hydrophobic wax barriers define a central sample addition zone and four auxiliary plasma collection zones containing dried reagents for quantification of glucose in plasma (Figure 11a). An input sample volume of only 7 μl of blood successfully filled the four plasma zones. Eliminating the void volume associated with a standard PSM layer for separation allowed lower volumes of blood to perform the same colorimetric assay. Similarly, a distance‐based μPAD featured the same Whatman 1 filter paper with a wax patterned sample addition zone and lateral channel (40 mm × 2 mm) to yield a dried plasma output.^127^ In this device, plasma separation was achieved by a hemostatic agent (3% w/v CaCO_3_/chitosan in 1 M acetic acid) stored in the sample addition zone, which induced clotting and blood aggregation by accelerating thrombin formation. The negative charge of blood cells, DNA, and some proteins electrostatically interacted with the positive charge of the chitosan material to physically trap blood cell aggregates. At the same time, calcium ions are required to promote clotting by transforming prothrombin to thrombin. Only a sample input of 3 μl whole blood was necessary for colorimetric quantitation of cardiac troponin 1 in a total of 15 min. Recently, a combination approach using agglutination antibodies and interlocked micropillar scaffolds (i.e., “synthetic paper”) was reported for separating plasma from whole blood with low protein adsorption.^126^ The device comprises a square‐shaped sample addition zone (16.8 mm) connected to a long rectangular channel (30 mm × 2 mm) for plasma separation and dried plasma output. Agglutination antibodies were spotted onto the sample addition zone to immobilize blood clots within the array of micropillars (50 μm diameter, spaced 100 μm apart) and allowed plasma to enter the lateral channel. An input volume of 90 μl whole blood was added and an average yield of 5–6 μl was obtained for a separation efficiency of 11.1%. Improved protein recovery was reported (>82%) in comparison to traditional PSM due to the low internal surface area of the material.

Traditional microfluidic devices have shown utility for plasma separation using a hand‐driven syringe or pipette for sample loading and collection to obviate external pumps. These devices yielded a liquid plasma output for immediate use for biochemical detection and immunoassay applications.^128^ However, previous examples suffered from slow separation, poor yield, and contamination from RBCs due to extended separation times.^129^, ^130^ A recent report aimed to address these limitations by integrating two sizes of microbeads with a capillary microchannel array for rapid separation of plasma and a liquid plasma output. A plasma separation filter was prepared by compactly stacking microbeads (10 and 100 μm diameters) into a polydimethylsiloxane microfluidic channel by negative pressure.^131^ The larger diameter beads were introduced first to block the openings of the capillary microchannels. Next, the smaller beads were stacked to retain the RBCs and allow liquid plasma to enter the array of microchannels. In this configuration, a separation rate of 0.16 μl min^−1^ was achieved with a sample input volume of 20 μl. Approximately 3.29 μl plasma was collected in 20 min. In comparison, the same channel stacked with 15 μm beads yielded a separation rate of only 0.03 μl min^−1^. While an improved separation rate was demonstrated using two sizes of stacked microbeads, this approach still suffers from lengthy separation times and poor yield.

Another separation mechanism using a microfluidic device employed constriction‐expansion channels for inertial cell sorting (Figure 11b).^132^ A narrow sample inlet constricted the blood cells into a tightly packed stream with a liquid plasma output at the periphery. Gradually increasing the dimensions of the channel created vortices (to trap cells) and skimming regions that directed liquid plasma to final collection zones. Plasma yield was evaluated as a function of expansion angle (5–20°) for undiluted whole blood. While this device performed independent of the hematocrit, only the 5° expansion angle produced sufficient separation. In this configuration, Shatova et al. achieved a flow rate of 50 μl min^−1^ using a hand‐driven syringe yielding 9% liquid plasma. Minimal hemoglobin was measured in the collected cell fractions and no ruptured RBCs were observed via microscopy.

The final example incorporated plasma separation and liquid plasma storage in porous superabsorbent polymer (PSAP) beads.^133^ These self‐driven microfilters comprise a system of interconnected pores, which form water channels upon hydration (Figure 11c). The small pore sizes (0.5–1 μm) effectively capture small analytes while excluding cells and bacteria. Swelling and absorption occurred relatively fast (5 min) for biofluids such as urine, however, undiluted whole blood required approximately 40 min to exclude roughly 88% of RBCs/WBCs. The PSAP beads exhibited a constant and precise swelling ratio (40–41 g g^−1^), which allowed the sample volume or weight to be calculated by directly counting the bead quantity. This essentially allowed pipet‐free aliquoting of biofluids. Excluding bacterial cells also reduced contamination at ambient or elevated temperatures thus eliminating the need for cold‐chain storage to maintain analyte stability. Chen et al. demonstrated excellent retention of catalase activity in hydrated PSAP beads at 35°C over a period of 7 days. Analytes were released from the PSAP beads using sonication. While nearly 12% of cells remained adsorbed to the surface of the beads, no cells were observed inside the channels. Prior to sonication, the beads could be washed to remove adsorbed cells as to not contaminate the final recovered liquid sample.

## 4 CONCLUSIONS

The development of miniaturized point‐of‐care technologies and hand‐held clinical analyzers has greatly increased testing capabilities outside of hospitals and clinics. However, pre‐analytical errors related to the quality and quantity of biological samples remains a considerable barrier. Specifically, the collection and preparation of whole blood and cell‐free plasma suffers from laboratory constraints requiring extensive infrastructure, reliable electricity, trained technicians, and cold‐chain storage. Innovative technologies designed for improved collection, transportation, and storage of whole blood have produced a wide range of user‐friendly tools with emphasis on volumetric techniques for more accurate and precise sampling. For applications where immediate on‐site testing is possible, liquid collection satisfies most analytical sampling requirements. When on‐site testing is not possible—or when archival samples are desired—storing dried blood or plasma in porous materials offers dual benefits of simple transportation by shipping through the mail and improved stability of myriad analytes at ambient conditions.

A remaining obstacle for expanding access to necessary healthcare diagnostics is the lack tools that offer multiple sample preparations within a single platform. In this review, we detailed materials, approaches, and novel technologies that yield a singular sample composition of either whole blood or cell‐free plasma. Some plasma separation technologies, in part, could feasibly generate unique samples containing plasma or blood cells by utilization of the separation membranes, but acceptable analytical quality of the cellular component has yet to be demonstrated. Applying the volumetric sampling techniques described here to passive methods of plasma separation could increase the diagnostic utility of the entire biological sample by streamlining collection efforts, reducing patient discomfort, and decreasing pre‐analytical errors. Overall, these challenges highlight both the significant roles that materials play in the collection, processing, and storage of blood to collect, and also the outstanding need for additional innovation in this space in order to continue to increase access to healthcare.

## AUTHOR CONTRIBUTIONS

**Keith R. Baillargeon:** Conceptualization (equal); data curation (lead); visualization (lead); writing – original draft (lead); writing – review and editing (lead). **Charles R. Mace:** Conceptualization (equal); funding acquisition (lead); supervision (lead); writing – review and editing (supporting).

## CONFLICT OF INTEREST

Keith R. Baillargeon and Charles R. Mace are co‐inventors on patent applications for technologies related to blood and plasma microsampling devices.

### PEER REVIEW

The peer review history for this article is available at https://publons.com/publon/10.1002/btm2.10476.

## Data Availability

No datasets were generated or analyzed during the preparation of this review.
